# Using instruments for selection to adjust for selection bias in Mendelian randomization

**DOI:** 10.1002/sim.10173

**Published:** 2024-07-22

**Authors:** Apostolos Gkatzionis, Eric J. Tchetgen Tchetgen, Jon Heron, Kate Northstone, Kate Tilling

**Affiliations:** ^1^ MRC Integrative Epidemiology Unit University of Bristol Bristol United Kingdom; ^2^ Population Health Sciences, Bristol Medical School University of Bristol Bristol United Kingdom; ^3^ Department of Statistics and Data Science, Wharton School University of Pennsylvania Philadelphia Pennsylvania

**Keywords:** ALSPAC, Heckman selection model, instrumental variables, Mendelian randomization, missing not at random, selection bias

## Abstract

Selection bias is a common concern in epidemiologic studies. In the literature, selection bias is often viewed as a missing data problem. Popular approaches to adjust for bias due to missing data, such as inverse probability weighting, rely on the assumption that data are missing at random and can yield biased results if this assumption is violated. In observational studies with outcome data missing not at random, Heckman's sample selection model can be used to adjust for bias due to missing data. In this paper, we review Heckman's method and a similar approach proposed by Tchetgen Tchetgen and Wirth (2017). We then discuss how to apply these methods to Mendelian randomization analyses using individual‐level data, with missing data for either the exposure or outcome or both. We explore whether genetic variants associated with participation can be used as instruments for selection. We then describe how to obtain missingness‐adjusted Wald ratio, two‐stage least squares and inverse variance weighted estimates. The two methods are evaluated and compared in simulations, with results suggesting that they can both mitigate selection bias but may yield parameter estimates with large standard errors in some settings. In an illustrative real‐data application, we investigate the effects of body mass index on smoking using data from the Avon Longitudinal Study of Parents and Children.

## 1 INTRODUCTION

Mendelian randomization (MR) uses genetic data to assess the causal relationship between a modifiable exposure and an outcome of interest.^1^, ^2^ MR is an application of instrumental variables analysis where genetic variants are used as instruments. The instrumental variable framework allows MR to account for unobserved confounding, which is a primary concern in other types of observational studies. However, just like any other epidemiological study, MR analyses remain susceptible to selection bias. There are several examples where this may occur. Studies of disease progression can be affected by selection bias, due to only observing disease progression traits on individuals who have developed the disease.^3^ Survivor bias can have an impact on MR analyses conducted on elderly cohorts. And large‐scale genetic datasets that are commonly used for MR, such as the UK Biobank, can yield biased results if the selection of participants into the dataset is not representative of the population from which they are selected.^4^, ^5^, ^6^

In this paper, we view selection bias as a missing data problem. Common approaches to adjust for missing data include inverse probability weighting (IPW) and multiple imputation; several authors have already considered the use of IPW to adjust for selection bias in MR.^7^, ^8^, ^9^, ^10^ These methods attempt to model the pattern of missingness or the distribution of missing values using fully observed variables, and thus rely on the assumption that data are missing at random (MAR). This assumption is questionable in some applications. For example, in the early days of the Covid‐19 pandemic, only individuals in high‐risk groups or those who exhibited Covid‐19 symptoms were tested for the virus. Asymptomatic patients from low risk groups were not tested and could not be classified as having Covid‐19. Therefore, in many Covid‐19 studies conducted in 2020, participation depended on developing Covid‐19‐related symptoms,^11^ and developing symptoms was in turn associated with Covid‐19 outcomes (eg, disease severity) resulting in data missing not at random (MNAR).

In this paper, we discuss how to adjust for selection bias in instrumental variable and Mendelian Randomization studies with individual‐level MNAR data. Our approach is based on Heckman's sample selection model,^12^ which was developed for observational studies in econometrics, as well as a recently proposed alternative method by Reference 13. The main idea is to identify a variable that is observed for all study participants and associated with selection into a study but not associated with the exposure and outcome of interest in the underlying population. Such a variable has been called an instrumental variable for selection, and can be used as an external source of information about the selection process, with which to adjust for selection bias. Despite being commonly used in econometrics, this approach has been underappreciated in genetic epidemiology.

We first present the method in the framework regression analyses in Section 2, starting from Heckman's original model for linear regression and then considering the more general setting of Tchetgen Tchetgen and Wirth. We then discuss our adaptation for MR studies in Section 3. Section 4 contains a range of simulations that were conducted to evaluate the two methods' performance, and in Section 5 we implement a real‐data application, assessing the effect of Body Mass Index (BMI) on smoking status and intensity, using data from the Avon Longitudinal Study of Parents and Children (ALSPAC).

## 2 INSTRUMENTS FOR SELECTION IN REGRESSION

### 2.1 Instrumental variable assumptions for selection

In this section, our interest lies in estimating the association between a vector of covariates X and an outcome Y, based on a sample $(X_{i},Y_{i}),i=1,\ldots ,n$. In particular, we consider the case where covariate values $X_{i}$ are observed for all individuals in the sample, but outcome values $Y_{i}$ are not. Let R be a selection indicator, such that if $R_{i}=1$ we observe $Y_{i}$ and if $R_{i}=0$ we do not. The observed data are $\left(X_{i},R_{i},R_{i}Y_{i}\right)$.

One approach to account for the missing outcome values in this setting is to use inverse probability weighting. IPW specifies a regression model for the selection indicator R and then weights each individual by the inverse of the probability of observing their outcome, in order to account for individuals with similar characteristics, for which the outcome is not observed. Standard IPW relies on a MAR assumption, which takes the form ℙ(R|X,Y)=ℙ(R|X) for the simple setting described above. If additional variables that affect missingness are available, these can be included in the model for R. Under the MAR assumption, if the weighting model is correctly specified, weighting by IPW is able to eliminate selection bias.

Now suppose that data are missing not at random (MNAR), whence ℙ(R|X,Y) depends on Y. Following the relevant literature, we let Z denote an additional variable that is observed for all individuals in the sample and is known to be associated with the selection indicator R. The variable Z is called an instrumental variable for selection if it satisfies two basic conditions:

1. Z⊥̸⊥R|X: the instrumental variable is associated with outcome missingness conditional on the observed covariates.
2. Z⊥⊥Y|X: the instrumental variable is independent of the outcome in the underlying population, conditional on observed covariates.

**FIGURE 1:**
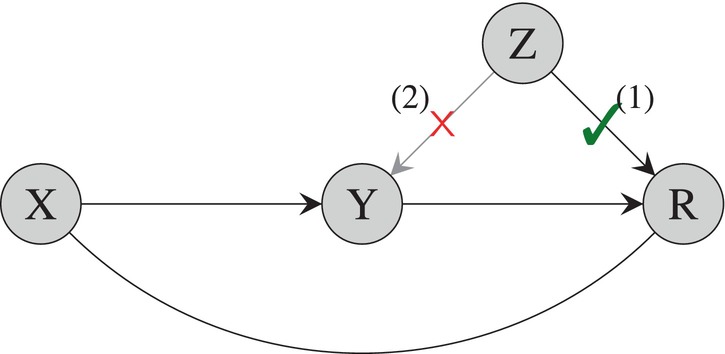
An illustration of the assumptions characterizing an instrumental variable for selection in observational studies.

These two assumptions are visualized in Figure 1. The first assumption resembles the relevance assumption of traditional instrumental variable analyses. The second assumption is similar to an exclusion restriction assumption; note that it does not prevent Z from exerting an effect on the covariates X or vice versa, nor does it prevent Z from affecting the outcome Y through its effect on X. Formally, the first assumption is testable but the second assumption is not. Together, they establish that Z can be used as an external source of information about the selection process, with which to adjust for selection bias.

### 2.2 Heckman's selection model

The idea of using an instrumental variable for selection in order to adjust for selection bias was first proposed by Heckman.^12^, ^14^ The modelling approach developed in these papers has become known as Heckman's sample selection model and is widely used in econometrics. Heckman considered the case of a normally distributed outcome,

(1) $$Y_{i}|X_{i}=X_{i}^{T}\beta +\epsilon_{i}\;,\;\;\;\;\;\epsilon_{i}∼N(0,\sigma_{1}^{2})$$

that is observed or missing according to a latent continuous process,

(2) $${\tilde{Y}}_{i}|{\tilde{X}}_{i}={\tilde{X}}_{i}^{T}\gamma +{\tilde{\epsilon }}_{i}\;,\;\;\;\;\;{\tilde{\epsilon }}_{i}∼N(0,\sigma_{2}^{2})$$

where $\tilde{X}$ represents variables that affect $\tilde{Y}$, and the error terms are correlated: $\text{Cov}(\epsilon_{i},{\tilde{\epsilon }}_{i})=\sigma_{12}$. The vector $\tilde{X}$ must include Z and may also include some of the covariates X or other fully observed variables. The inclusion of Z in (2) but not in (1) is important to avoid issues of collinearity when fitting the model.^15^

In Heckman's model, the outcome $Y_{i}$ is observed for individual i if and only if ${\tilde{Y}}_{i}>0$, which is equivalent to a probit selection model. The objective of inference is to estimate the parameter vector β. Heckman showed that

(3) $$𝔼(Y_{i}|X_{i},{\tilde{Y}}_{i}>0)=X_{i}^{T}\beta +\frac{\sigma_{12}}{\sigma_{2}}\lambda_{i}$$

where $\lambda_{i}$ is called the inverse Mills ratio and satisfies $\lambda_{i}=\frac{\phi (\Lambda_{i})}{1-\Phi (\Lambda_{i})}$, $\Lambda_{i}=-\frac{{\tilde{X}}_{i}\gamma }{\sigma_{2}}$, using ϕ, Φ to denote the density function and cumulative distribution function of a N(0,1) random variable respectively. The expectation $𝔼(Y_{i}|X_{i},{\tilde{Y}}_{i}>0)$ is taken over the observed data, while the inverse Mills ratio can be computed from the probit selection model. Therefore, Equation (3) provides a way of estimating the full‐data regression parameter β using the observed data alone.

Heckman proposed a two‐stage procedure in order to fit model (1) and (2). In the first step, probit regression is used to model the selection indicator $R=\{\tilde{Y}>0\}$ in terms of fully observed covariates $\tilde{X}$. The fitted values from this regression are then used to estimate the inverse Mills ratios $\lambda_{i}$ for all individuals in the sample. In the second step, the outcome is regressed on the covariates $X_{i}$ and the inverse Mills ratios, using only data on individuals with observed outcome values. A parameter estimate for the regression coefficient β can be obtained from this second‐stage regression; we refer to Heckman's work for details on how to compute its standard error. Alternatively sample selection models can be fitted using maximum likelihood estimation.^16^

### 2.3 The method of Tchetgen Tchetgen and Wirth

Heckman's sample selection model makes fairly restrictive parametric assumptions, requiring a normally distributed outcome and a probit model for the selection process. Various authors have attempted to relax these assumptions; we provide a brief literature review in the Supplementary Material. In this paper, we focus on an extension proposed by Reference 13. As previously, let Z be an instrumental variable associated with the selection indicator R but not with the outcome Y conditional on observed covariates. To guarantee parameter identification across a wide range of statistical models, Tchetgen Tchetgen and Wirth impose the assumption that selection bias must be homogeneous on the scale of the parameter of interest. The exact mathematical form of the assumption depends on the model that is fitted; TTW considered linear, logistic and Poisson regression as examples. Here, we illustrate the assumption and estimation process for linear regression and delegate a discussion of logistic and Poisson regression to the Supplementary Material.

Suppose that a continuous outcome Y is modelled using linear regression (1), as in Heckman's work. In this setting, selection bias can be quantified as the mean difference in outcome values between individuals with observed and unobserved outcomes:

(4) 𝔼(Y|X,Z,R=1)−𝔼(Y|X,Z,R=0)=δ(X,Z)

The homogeneous selection bias assumption states that this bias is only a function of the observed covariates and does not depend on the instrument: δ(X,Z)=δ(X). Intuitively, this means that the instrument for selection should affect the chances of observing an individual participant's outcome but should not affect the overall magnitude of selection bias in the analysis.

As demonstrated in Heckman's work, identification in linear regression is a consequence of the model assumptions and the homogeneous selection bias assumption is not necessary. However, the assumption can be used to derive a formula for estimation, similar to (3):

(5) 𝔼(Y|X,Z,R=1)=𝔼(Y|X)+δ(X)(1−π(X,Z))

where $𝔼(Y|X)=X^{T}\beta$ in the case of linear regression. The function π(X,Z)=ℙ(R=1|X,Z) represents the propensity score for the selection indicator given X and Z. Formula (5) links the full‐data regression 𝔼(Y|X) with the observed‐data regression 𝔼(Y|X,Z,R=1) and can be used to construct a likelihood for Y|X,Z,R=1. For linear regression with normally distributed errors, the likelihood has the form

$$ℒ(Y|X,Z,R=1)=\overset{n}{\underset{i=1}{\prod }}\left[{\left(\phi (Y_{i};X_{i}^{T}\beta +\delta (X_{i})(1-\pi (X_{i},Z_{i})),\sigma^{2})\right)}^{R_{i}}f_{B}(R_{i};\pi (X_{i},Z_{i}))\right]$$

Here, $\phi (x;\mu ,\sigma^{2})$ is the normal density function and $f_{B}(x;\pi )$ is the Bernoulli probability mass function. If one is willing to make a set of parametric assumptions for the propensity score π(X,Z) and the selection bias function δ(X), for example

$$\delta (X)=X^{T}\eta \;\;\;,\;\;\;\pi (X,Z)={\left(X\;\;Z\right)}^{T}\alpha$$

then the likelihood can be maximized to yield consistent parameter estimates for the parameter vector $\left(\beta ,\eta ,\alpha ,\sigma^{2}\right)$. Maximization can be implemented either as standard optimization over all the parameters simultaneously, or as a partial optimization procedure where one first maximizes the likelihood for the propensity score, $\prod_{i=1}^{n}\left(f_{B}(R_{i};\pi (X_{i},Z_{i}))\right)$, and then maximizes the likelihood for the outcome using the fitted values for the propensity score parameters. Although less flexible and less efficient in principle, the partial optimization procedure can have computational advantages in practice. Standard error estimates can be derived from the Hessian matrix at the optimal parameter values, as is common with maximum likelihood procedures.

The TTW method is not restricted to linear regression and can be applied to more general models, either by relaxing the linearity assumption and considering non‐linear (and possibly non‐parametric) models where 𝔼(Y|X)=μ(X;β), or by modelling non‐normal outcomes e.g. via logistic or Poisson regression. The homogeneous selection bias assumption may not be strictly necessary for simple parametric models like linear regression, but becomes necessary for identification if the regression model is made flexible enough. We expand on these points in the Supplementary Material.

## 3 ADJUSTING FOR SELECTION BIAS IN MENDELIAN RANDOMIZATION

### 3.1 Missing data in MR studies

We now discuss how to utilize “instruments for selection” methods and adjust for selection bias due to MNAR data in Mendelian randomization studies. Some results in this direction have been reported in the econometrics literature.^17^ We will use the abbreviation “IVsel” to refer to the “instruments for selection” methods, including both Heckman's sample selection and the TTW method.

In this section, we let X denote the exposure and Y the outcome of the MR analysis. Our objective is to infer the causal relationship between X and Y, using a genetic instrument G to account for the presence of unmeasured confounders U. We will assume that G satisfies the traditional instrumental variable assumptions (is associated with X, is independent of U, and does not affect Y by any other causal pathway except through X), and hence it is a valid instrument for inference in the absence of missing data.

Missing data can affect the MR analysis in three ways: data can be missing for the exposure but not the outcome, or for the outcome but not the exposure, or for both the exposure and the outcome. Each of these three scenarios can induce selection bias. For example, in an MR study with missing exposure values, if selection is only affected by the outcome Y, the data will be missing at random and selection bias will be eliminated by applying IPW. However, if the exposure X also affects selection into the study, the data will be missing not at random and it will not be possible to fully model the selection process using the observed data alone.

### 3.2 Instruments for selection in MR

**FIGURE 2:**
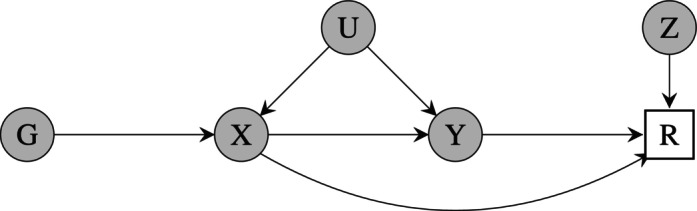
A directed acyclic graph depicting the use of instruments for selection in Mendelian randomization. In this graph, both the exposure and the outcome are assumed to affect selection into the study.

To account for selection bias, we leverage an instrumental variable Z for the selection process, satisfying the IV assumptions of Section 2.1. The value of the instrument Z must be observed for all individuals in the sample, including those with missing exposure and/or outcome measurements. Figure 2 illustrates how instruments for selection may be used to adjust for selection bias in MR studies.

The instrument for selection can be either genetic or non‐genetic. Applications of Heckman's model in econometrics and social sciences have so far relied on non‐genetic instruments. For example, when working with questionnaire data, potential instrumental variables can include interviewer characteristics (eg, gender) or the mode of contact of study participants (by phone, email, etc.). At the same time, when applying the IVsel methods in genetic epidemiology, it may be possible to obtain a genetic instrument for selection. This would require genetic information on both selected and unselected individuals to be available, so that genetic variants associated with selection (but not the exposure and/or outcome) can be identified. For example, Reference 18 identified genetic variants associated with participation in optional participation components of the UK Biobank dataset. These variants can be used as instruments in MR studies of exposures or outcomes from UK Biobank's optional participation components, in order to adjust for selection bias due to optional participation. As another example, in MR studies of disease progression, a genetic instrument for selection can be constructed using genetic variants associated with disease incidence but not with disease progression.

### 3.3 MR with a single instrument for inference

We now discuss how the IVsel methods can be implemented in MR, when individual‐level data are available. First, we consider Mendelian randomization analyses using a single instrument for inference. This can be either a single genetic variant or an allele score comprising of multiple variants. Consider the simple MR model

(6) $$\begin{array}{ll} X_{i} & =\beta_{X}G_{i}+\epsilon_{Xi}\\ Y_{i} & =\theta X_{i}+\epsilon_{Yi}\\ & =\theta \beta_{X}G_{i}+(\theta \epsilon_{Xi}+\epsilon_{Yi})\\ & =\beta_{Y}G_{i}+\epsilon_{Yi}^{\prime } \end{array}$$

with $\left(\epsilon_{Xi},\epsilon_{Yi})∼N(0,\sum \right)$. The causal effect parameter θ is usually estimated by the Wald ratio,

(7) $$\hat{\theta }=\frac{{\hat{\beta }}_{Y}}{{\hat{\beta }}_{X}}\;,\;\;\;\text{s.e.}\left(\hat{\theta }\right)=\sqrt{\frac{{ŝ}_{Y}^{2}}{{\hat{\beta }}_{X}^{2}}+\frac{{\hat{\beta }}_{Y}^{2}{ŝ}_{X}^{2}}{{\hat{\beta }}_{X}^{4}}}$$

using a second‐order approximation for the standard error. With individual data, the Wald ratio can be computed by regressing the exposure X and outcome Y respectively on G, and then taking the ratio of the regression coefficients from the two fits. The ordinary least squares (OLS) estimates ${\hat{\beta }}_{X}$, ${\hat{\beta }}_{Y}$ are consistent for $\beta_{X}$, $\beta_{Y}$, and provided that $\beta_{X}\neq 0$ (which is guaranteed by the IV assumptions), their ratio will be a consistent estimate of the causal effect θ. This estimation strategy remains valid when the outcome Y is binary.^19^

With MNAR missing data for the exposure and/or outcome, the OLS estimates are biased. However, if an instrument for selection is available, Heckman's sample selection model or the TTW maximum likelihood procedure can be used to obtain asymptotically unbiased estimates of $\beta_{X}$, $\beta_{Y}$ and their standard errors. This needs to be done only for the variable for which there are missing data; for example, in a study where data are only missing for the outcome Y, the G−X association can still be estimated by OLS. The estimates ${\hat{\beta }}_{X},{\hat{\beta }}_{Y}$ can then be combined using the Wald ratio. The fact that this ratio is an asymptotically unbiased estimate of the X−Y causal effect follows directly from standard MR theory.

Regarding the IV assumptions for selection, the relevance assumption extends straightforwardly to MR studies and requires the instrument to be associated with selection into the study. The exclusion restriction assumption effectively requires the instrument to be independent of any variable for which data are missing. In an MR study with missing data only for the exposure, Z must be independent of the exposure (conditional on the genetic instrument G for inference) in order to adjust for selection bias when estimating the G−X association. However, Z can be associated with Y without violating the exclusion restriction, since the G−Y association can be estimated using OLS. In an MR study with missing outcome data, the instrument for selection must be independent of the outcome (conditional on G). In this case, a Z−X association is also a violation of the exclusion restriction assumption, because, if X is causal for Y, an association between the instrument and the exposure will open a pathway between Z and Y that is not mediated by G, and will induce bias in computing the G−Y association estimate. Finally, in MR studies with missing data for both the exposure and the outcome, it follows trivially that the instrument for selection must be independent of both X and Y in the underlying population.

### 3.4 MR with multiple instruments

In practice, Mendelian randomization is often conducted using multiple instrumental variables for inference. A simple MR model with K genetic instruments is

(8) $$\begin{array}{ll} X_{i} & =G_{i}^{T}\beta_{X}+\epsilon_{Xi}\\ Y_{i} & =\theta X_{i}+\epsilon_{Yi}\\ & =G_{i}^{T}\beta_{Y}+\epsilon_{Yi}^{\prime }\\ \left(\epsilon_{Xi},\epsilon_{Yi}\right) & ∼N(0,\sum ) \end{array}$$

where G represents a n×K genetic data matrix and $\beta_{X},\beta_{Y}$ are vector‐valued. Common approaches for MR include two‐stage least squares (2SLS) estimation when working with one‐sample individual‐level data, and the inverse variance weighted (IVW) formula when working with two‐sample data. We will consider these two approaches separately and discuss how they can be combined with the IVsel methods.

#### 3.4.1 Two‐stage least squares

The 2SLS procedure consists of first regressing the exposure X on genetic variants $G_{1},\ldots ,G_{K}$ and obtaining fitted exposure values $\hat{X}$, and then regressing the outcome Y on the fitted values $\hat{X}$ and using the regression coefficient as an estimate of the causal effect θ. The method requires access to one‐sample individual‐level data. Extensions of 2SLS can handle non‐normal or non‐linear exposure‐outcome relationships by using fitted first‐stage residuals instead of exposure values.^20^

2SLS can be readily combined with IVsel approaches. With missing data for the exposure, the first‐stage regression can be replaced by an implementation of the methods of Heckman or TTW. This will yield consistent G−X association estimates, and hence fitted exposure values that are free of selection bias. The second‐stage regression can be implemented as usual. With missing outcome data, instruments for selection can be used as part of the regression of Y on $\hat{X}$ instead. In both cases, the causal effect estimates retain the consistency properties of the original 2SLS method. However, the same cannot be said about the estimated standard errors. In traditional 2SLS regression, the standard error associated with ${\hat{\theta }}_{\text{2SLS}}$ cannot be computed from the second‐stage regression alone, as it needs to be adjusted for uncertainty in the first stage. The formula to compute the standard error is

(9) $$\begin{array}{ll} \text{s.e.}\left({\hat{\theta }}_{\text{2SLS}}\right) & ={\left(X^{T}G{\left(G^{T}G\right)}^{-1}G^{T}X\right)}^{-1}{\hat{\sigma }}^{2}\\ {\hat{\sigma }}^{2} & =\frac{1}{n-1}{\left(Y-X\theta \right)}^{T}(Y-X\theta ) \end{array}$$

This formula cannot be used in conjunction with the IVsel approaches. With missing data for the exposure, the vector X is only partially observed and the standard error cannot be computed from (9). On the other hand, with missing outcome data, the standard error (9) can be computed but the estimator to which it corresponds is biased. Instead, an unbiased estimate of the 2SLS standard error can be obtained using a bootstrap algorithm:

1. Given data on individuals i=1,…,N, repeat the following procedure M times (j=1,…,M):
  - Sample individuals $(i_{1}^{\left(j\right)},\ldots ,i_{N}^{\left(j\right)})\in \left\{1,\ldots ,N\right\}$ with replacement.
  - For the sampled individuals, run 2SLS using Heckman's method or TTW to adjust for missing exposure or outcome data.
  - Obtain a sample‐specific causal effect estimate ${\hat{\theta }}^{\left(j\right)}$.
2. Use the standard deviation of the M values ${\hat{\theta }}^{\left(j\right)}$, j=1,…,M as an estimate of the 2SLS standard error.

However, this can be computationally intensive, especially when combined with the maximum likelihood procedure of the TTW method. With large numbers of genetic variants, a computationally efficient alternative could be to combine genetic variants into a single allele score and then use a Wald ratio estimate for the allele score.

#### 3.4.2 Selection‐adjusted summary statistics

In practice, MR is often conducted using a two‐sample framework in which genetic associations with the exposure and outcome are estimated in different datasets. This is particularly common when working with summary‐level data. For genetic variant $G_{j}$, j=1,…,K, let ${\hat{\beta }}_{Xj}$, ${\hat{\beta }}_{Yj}$ denote its association with the exposure and outcome respectively, and let ${\hat{\sigma }}_{Xj}^{2}$, ${\hat{\sigma }}_{Yj}^{2}$ denote the corresponding standard errors. The causal effect θ can be estimated from the inverse variance weighted formula,

(10) $${\hat{\theta }}_{IVW}=\frac{\sum_{j}{\hat{\beta }}_{Xj}{\hat{\beta }}_{Yj}{\hat{\sigma }}_{Yj}^{-2}}{\sum_{j}{\hat{\beta }}_{Xj}^{2}{\hat{\sigma }}_{Yj}^{-2}}\;,\;\;\;\text{s.e.}\left({\hat{\theta }}_{IVW}\right)=\frac{1}{\sqrt{\sum_{j}{\hat{\beta }}_{Xj}^{2}{\hat{\sigma }}_{Yj}^{-2}}}$$

With access to individual‐level data, the summary statistics can be computed from a sequence of univariate linear regressions for each genetic variant separately, and then combined using IVW. In the presence of missing data, IVsel methods can be used to adjust the computation of summary statistics in a similar way as for MR with a single genetic instrument. The adjustment needs to be implemented for each genetic variant separately, meaning that the IVsel methods have to be implemented K times, but the computational cost of doing so is not prohibitive. This procedure yields a set of “selection‐adjusted summary statistics" that can then be combined using the IVW formula (10). Since estimates from the IVsel methods are consistent and asymptotically normal, this selection‐adjusted IVW estimate will retain its standard theoretical properties.

The IVW estimator requires summary statistics ${\hat{\beta }}_{Xj}$, ${\hat{\beta }}_{Yj}$ to come from non‐overlapping samples, therefore it is most commonly used in a two‐sample MR setting. At the same time, the implementation of IVsel methods requires individual‐level data. Therefore, the procedure described above is most efficient when having access to two separate individual‐level datasets. However, gaining access to individual‐level data from two separate sources can be difficult in practice. In the more common scenario of a single individual‐level dataset, IVsel methods can still be combined with IVW but one has to guard against potential bias due to sample overlap. The effect of sample overlap is to exacerbate weak instrument bias.^21^ Therefore, we would recommend that the IVsel methods are only combined with IVW in applications where the instruments for inference are strong and weak instrument bias is of lesser concern. Alternatively, the selection‐adjusted summary statistics can be used in conjunction with recently proposed approaches to remove bias due to sample overlap.^22^, ^23^

Individual‐level data may not be necessary for both datasets in the two‐sample setting. For example, in a two‐sample MR study of disease progression, individual‐level data will be needed for the disease progression trait in order to implement IVsel methods and adjust for selection bias due to conditioning on disease incidence. However, genetic associations with the exposure can be estimated from a summary‐level dataset.

Access to individual‐level data allows for estimating joint genetic effects, as well as marginal ones. This makes it straightforward to adjust for genetic correlations in MR analyses with correlated genetic variants. The two IVsel methods can be implemented with multiple covariates, meaning that modelling correlated variants remains straightforward in studies affected by sample selection.

Finally, recent years have seen the development of a plethora of MR methods using summary statistics, including adjustments for pleiotropic bias, for example, References 24, 25, 26, 27, 28 and multivariable MR,^29^, ^30^ among other topics. In principle, selection‐adjusted summary statistics can facilitate the use of such methods in applications with individual‐level data and non‐random missingness. However, this goes beyond the scope of the current paper.

## 4 SIMULATION STUDY

We implemented a simulation study to explore the performance of IVsel approaches in adjusting for selection bias. Simulations were conducted both for regression analyses, where the aim was to estimate the statistical association between a covariate X and an outcome Y, and in a MR setting where the parameter of interest was the X−Y causal effect. Although our focus in this paper is on applications to instrumental variable and MR analyses, we also conduct simulations for regression analyses. This is done for two reasons. First, the finite‐sample performance of Heckman's sample selection model has been investigated in the past, but the relevant literature for the more recent TTW method has been limited. Second, MR estimates are derived as a combination of two regression‐based estimates (eg, the G−X and G−Y associations for a Wald ratio, or the two models fitted as part of a 2SLS algorithm), and adjusting for selection will affect MR estimates via these models. Thus it is important to understand how the IVsel methods perform in the regression framework in order to investigate their performance in MR analyses.

### 4.1 Regression analyses

#### 4.1.1 Simulation design

First, we considered regression analyses. We assumed the existence of a single covariate X, whose association with the outcome Y needs to be estimated. Keeping the notation consistent with Section 2, our baseline simulation setting was as follows:

$$\begin{array}{ll} Z,X,\epsilon & ∼N(0,1)\;\;\;\text{independently}\\ Y & =\alpha +\beta X+\epsilon \\ R & ∼\text{Logistic}(\pi_{R})\\ \text{logit}\left(\pi_{R}\right) & =\alpha_{R}+\beta_{R}X+\gamma_{R}Z+\delta_{R}Y \end{array}$$

The parameter of interest was the regression coefficient β. In our baseline simulation, we set β=0.1, with this value chosen to facilitate a power comparison. The intercept α was set equal to 1. The parameter $\gamma_{R}$ determines instrument strength and was set to $\gamma_{R}=0.4$ which corresponds to an $R^{2}$ statistic of 0.02 for the instrument Z. The selection effects were set equal to $\beta_{R}=0.5$ and $\delta_{R}=0.5$, corresponding to an $R^{2}$ statistic of about 0.08 for the covariate and outcome combined. Finally, the value of the intercept $\alpha_{R}$ was chosen to ensure that approximately 50% of individuals had missing outcome data (in this baseline simulation, this meant that $\alpha_{R}=-\alpha \delta_{R}=-0.5$). The simulation was implemented for $N=10^{4}$ individuals.

We then considered the following modifications of our baseline scenario:

- A null regression coefficient (β=0).
- A binary instrument Z.
- A binary covariate X.
- A binary outcome (generated according to a logistic regression model).
- A discrete outcome (generated according to a Poisson regression model).
- Selection affected by the outcome but not the covariate.
- Selection affected by the covariate but not the outcome.
- A direct Z−X effect.
- A direct (“pleiotropic”) Z−Y effect.

The direct Z−X and Z−Y effects were specified by modifying the generation of exposure and outcome values to be $X=\beta_{X}Z+\epsilon_{X}$, $\epsilon_{X}∼N(0,1)$ and Y=α+βX+γZ+ϵ, ϵ∼N(0,1) respectively; for these simulations we set $\beta_{X}=\frac{1}{3}$ and $\gamma =\frac{1}{3}$, which correspond to an instrument Z that explained approximately 10% of variation in the exposure or outcome.

Along with our baseline scenario, there were a total of 10 simulation settings. Each simulation was replicated $10^{4}$ times.

In a second simulation, we explored how the performance of IVsel methods was affected by the strength of the instrument for selection. In our simulation, instrument strength is determined by the parameter $\gamma_{R}$. We varied the value of that parameter, setting it equal to 0, 0.08, 0.2, 0.27, 0.4, 0.6 and 0.95. These values were selected to yield $R^{2}$ statistics of 0, 0.1%, 0.5%, 1%, 2%, 5% and 10% respectively. Note that the $R^{2}$ statistic models the proportion of variation in the selection process that is explained by the instrument Z in each simulation. All other aspects of the simulation design were kept the same as in our baseline scenario.

In each simulation scenario, we implemented Heckman's sample selection and the TTW method to estimate the regression coefficient β. We also implemented complete‐case analysis (CCA), using only information on individuals with observed outcome values, as well as an inverse probability weighting (IPW) approach. These two methods rely on the MAR assumption and are expected to yield biased results in our simulations. Finally, we also computed “oracle” estimates using both observed and missing data, as a benchmark for comparison.

For the IPW method, we computed the inverse probability weights using a logistic regression model without interactions, which was the true model for R in our simulation. We included the exposure X but not the instrument Z as explanatory variables in the logistic model; note that Z is independent of the outcome, therefore its inclusion would offer little to no benefit to the IPW method.^31^ The method was used simply as a benchmark for comparison with the IVsel methods in our simulations. As such, our implementation of IPW was quite simplistic; for example, we did not consider flexible weighting models and did not implement doubly robust estimation.^32^ These extensions can improve the performance of IPW in analyses with MAR data but would not be able to offer consistent estimation under our MNAR setting. For Heckman's method we included both Z and X in its selection model. For the TTW method, we used partial likelihood optimization; an implementation using the full likelihood yielded similar results. The method was implemented using a logistic model for the propensity score. It is possible to implement the method using a more flexible non‐parametric propensity score model; indeed, this is one of the method's advantages over Heckman's approach. However, we have decided to use the logistic model here for simplicity. Standard errors were computed by inverting the (negative) Hessian matrix evaluated at the optimal parameter values and then taking the square roots of the diagonal elements, as is common with maximum likelihood‐type methods. All simulations were conducted in the statistical software R. Heckman's method was implemented using the R package sampleSelection.^33^ Standard errors for IPW were computed using the survey package.^34^ The other methods were coded by hand. Our R code and simulation results are available at https://github.com/agkatzionis/IVsel.

#### 4.1.2 Simulation results

Simulation results are reported in Table 1 for the 10 exploratory scenarios and in Table 2 for the simulations with varying instrument strength. For each method, we report parameter estimates for the regression coefficient β, the empirical standard deviation of estimates across replications, model‐based standard errors, the empirical coverage of 95% confidence intervals and the empirical power to reject the null hypothesis $H_{0}:\;\beta =0$ (except in the scenario where β=0).

**TABLE 1:** Performance of selection bias adjustment methods in a range of different simulation scenarios.

Method	Mean	Emp SD	StdErr	Cover	Power	Mean	Emp SD	StdErr	Cover	Power
	Baseline scenario					β=0				
CCA	0.045	0.014	0.014	0.033	0.888	−0.050	0.014	0.014	0.059	—
IPW	0.045	0.011	0.011	0.001	0.984	−0.050	0.010	0.011	0.003	—
Heckman	0.099	0.024	0.024	0.952	0.989	0.000	0.022	0.022	0.950	—
TTW	0.102	0.045	0.044	0.948	0.642	0.002	0.046	0.045	0.948	—
Oracle	0.100	0.010	0.010	0.953	1.000	0.000	0.010	0.010	0.951	—
	Binary Z					Binary X				
CCA	0.045	0.014	0.014	0.029	0.890	−0.009	0.028	0.028	0.029	0.058
IPW	0.045	0.011	0.011	0.000	0.982	−0.008	0.020	0.021	0.000	0.060
Heckman	0.099	0.023	0.023	0.953	0.989	0.099	0.047	0.047	0.952	0.557
TTW	0.102	0.045	0.044	0.946	0.639	0.110	0.174	0.175	0.951	0.098
Oracle	0.100	0.010	0.010	0.949	1.000	0.100	0.020	0.020	0.949	0.999
	Binary outcome					Discrete outcome				
CCA	−0.009	0.038	0.038	0.176	0.058	0.071	0.008	0.008	0.071	1.000
IPW	−0.007	0.024	0.025	0.009	0.053	0.071	0.007	0.007	0.008	1.000
Heckman	0.055	0.032	0.032	0.721	0.418	—	—	—	—	—
TTW	0.100	0.123	0.116	0.909	0.181	0.101	0.023	0.023	0.949	0.991
Oracle	0.100	0.023	0.023	0.949	0.994	0.100	0.006	0.006	0.947	1.000
	No X−R effect					No Y−R effect (MAR)				
CCA	0.089	0.013	0.013	0.878	1.000	0.100	0.015	0.015	0.948	1.000
IPW	0.090	0.009	0.010	0.836	1.000	0.100	0.012	0.012	0.951	1.000
Heckman	0.100	0.014	0.014	0.952	1.000	0.100	0.030	0.029	0.946	0.919
TTW	0.101	0.076	0.075	0.946	0.274	0.099	0.042	0.041	0.948	0.675
Oracle	0.100	0.010	0.010	0.952	1.000	0.100	0.010	0.010	0.951	1.000
	Added Z−X effect					Added Z−Y effect				
CCA	0.035	0.014	0.014	0.004	0.727	0.029	0.015	0.015	0.002	0.497
IPW	0.034	0.011	0.011	0.000	0.872	0.028	0.011	0.012	0.000	0.681
Heckman	0.098	0.028	0.027	0.946	0.950	−0.218	0.024	0.024	0.000	1.000
TTW	0.103	0.041	0.040	0.947	0.721	−0.183	0.042	0.039	0.000	0.995
Oracle	0.100	0.010	0.009	0.949	1.000	0.100	0.011	0.011	0.951	1.000

**TABLE 2:** Performance of selection bias adjustment methods in simulations with varying degrees of instrument strength for the instrument for selection.

Method	Mean	Emp SD	StdErr	Cover	Power	Mean	Emp SD	StdErr	Cover	Power
	$R^{2}=0.1\%$					$R^{2}=0.5\%$				
CCA	0.042	0.014	0.014	0.017	0.841	0.043	0.014	0.014	0.019	0.860
IPW	0.042	0.011	0.011	0.001	0.968	0.043	0.011	0.011	0.001	0.973
Heckman	0.094	0.090	0.090	0.966	0.167	0.099	0.040	0.040	0.956	0.703
TTW	0.106	0.106	0.102	0.950	0.186	0.102	0.056	0.056	0.948	0.450
Oracle	0.100	0.010	0.010	0.952	1.000	0.100	0.010	0.010	0.952	1.000
	$R^{2}=1\%$					$R^{2}=2\%$				
CCA	0.044	0.014	0.014	0.021	0.874	0.045	0.014	0.014	0.033	0.888
IPW	0.043	0.011	0.011	0.000	0.976	0.045	0.011	0.011	0.001	0.984
Heckman	0.099	0.031	0.031	0.951	0.892	0.099	0.024	0.024	0.952	0.989
TTW	0.103	0.051	0.050	0.949	0.543	0.102	0.045	0.044	0.948	0.642
Oracle	0.100	0.010	0.010	0.949	1.000	0.100	0.010	0.010	0.953	1.000
	$R^{2}=5\%$					$R^{2}=10\%$				
CCA	0.049	0.014	0.014	0.049	0.933	0.057	0.014	0.014	0.137	0.979
IPW	0.049	0.011	0.011	0.002	0.994	0.056	0.011	0.011	0.018	1.000
Heckman	0.100	0.018	0.019	0.955	0.999	0.100	0.016	0.016	0.952	1.000
TTW	0.102	0.039	0.039	0.949	0.742	0.101	0.033	0.032	0.946	0.878
Oracle	0.100	0.010	0.010	0.949	1.000	0.100	0.010	0.010	0.952	1.000

Our results for the baseline simulation scenario suggest that IVsel methods were able to adjust for selection bias due to missing data and returned unbiased estimates of the regression coefficient. The associated standard errors were fairly high compared to oracle estimates, resulting in low power to reject the null hypothesis $H_{0}:\;\beta =0$, but the methods maintained nominal coverage and Type I error rates. Between the two methods, estimates from Heckman's method had smaller standard errors compared to the TTW method. IPW and complete‐case analysis exhibited moderate degrees of bias, as expected.

Similar results were obtained in the simulation with β=0. Note that in this scenario, the coverage figures reported in Table 1 equal one minus the Type I error rate, suggesting that IVsel methods attain nominal Type I error rates under a null regression coefficient.

For a binary instrument, IVsel methods were again able to adjust for selection bias while producing confidence intervals with nominal coverage. For a binary covariate, Heckman's method exhibited good performance but the results obtained from the TTW method were subject to very high uncertainty. For a binary outcome, Heckman's method exhibited a small degree of downward bias, while the TTW method obtained unbiased point estimates but exhibited slight undercoverage. In general, we have found the implementation of Heckman sample selection models in R to be less efficient for binary outcomes than for continuous ones. Nevertheless, both IVsel methods performed better than complete‐case analysis and IPW. For a discrete outcome, the existing implementation of Heckman's method in R does not support fitting a Poisson model,^35^ therefore results from this method are not reported. The performance of the TTW method was satisfactory.

In simulations where selection was only a function of the outcome and not the covariate, the MAR methods remained biased but the bias was markedly smaller. The TTW approach resulted in higher standard errors compared to the baseline scenario, while Heckman's method produced smaller standard errors. This is further explored in Supplementary Material. The performance of all methods improved further in simulations where only the covariate, and not the outcome, affected selection. Since the covariate was fully observed in our simulations, this scenario represents a MAR mechanism, meaning that IPW can fully account for selection bias; indeed, the method exhibited no bias and nominal coverage in our simulations. Complete‐case analysis was also unbiased, because the probability of observing the outcome was independent of the value of the outcome conditional on X.^36^

IVsel methods were able to accommodate an effect of the instrument on the covariate, as it does not violate the instrumental variable assumptions for Z. On the other hand, a direct Z−Y effect constitutes a violation of the exclusion restriction assumption, and this induced bias in parameter estimates obtained from the IVsel methods. The magnitude of bias depends on the strength of the Z−Y association, but our simulations indicate that the bias can be quite severe.

Table 2 contains results from our simulations exploring the performance of IVsel methods for varying degrees of instrument strength. The IVsel estimates were unbiased and attained nominal coverage regardless of the $R^{2}$ value, but weaker instruments led to more uncertainty about parameter estimates and lower power. The lack of bias is noteworthy: in MR analysis, using a weak instrument for inference will typically induce weak instrument bias in causal effect estimates. This happens because the MR estimand is effectively a ratio, and high uncertainty about the denominator of the ratio will not affect the value of the estimand in a symmetric way. On the other hand, in the simulations of Table 2, the estimand is a single parameter, and a weak instrument will produce a parameter estimate with high uncertainty but no bias.

Strong instruments resulted in IVsel estimates with high precision. When instrument strength was 5%–10%, the accuracy of Heckman's sample selection method was comparable to that of IPW, while the TTW method exhibited more uncertainty than other approaches but had reasonable power. The increased uncertainty of the TTW method is not surprising, given that the method makes rather flexible modelling assumptions, but may also be a consequence of the misspecification of the propensity model π(X,Z) (the IVsel methods assume a probit model but we used a logistic model in our simulations). Note that the TTW method is likely to be more sensitive to such misspecification than Heckman's algorithm, since correct specification of π(X,Z) is necessary to establish identification.

Overall, this simulation suggests that although IVsel methods can yield valid inferences with weak instruments, it is still important to identify strong instruments when using the methods in practice.

### 4.2 Mendelian randomization

#### 4.2.1 Simulation design

In our MR simulations, the aim was to estimate the causal effect of an exposure X on an outcome Y in the presence of missing (MNAR) data for the exposure and/or outcome. We implemented two sets of MR simulations, one using a single genetic instrument for inference and a second one using multiple instruments. For the simulations with a single instrument for inference, we used the following data‐generating model:

$$\begin{array}{ll} G,Z,U,\epsilon_{X},\epsilon_{Y} & ∼N(0,1)\\ X & =\alpha_{X}+\beta_{X}G+\gamma_{X}U+\epsilon_{X}\\ Y & =\alpha_{Y}+\theta X+\gamma_{Y}U+\epsilon_{Y}\\ R & ∼\text{Logistic}(\pi_{R})\\ \text{logit}\left(\pi_{R}\right) & =\alpha_{R}+\beta_{R}X+\gamma_{R}Z+\delta_{R}Y \end{array}$$

The genetic instrument G was generated according to a normal distribution to represent an MR analysis where a polygenic risk score is used a an instrumental variable.

In simulations with multiple instruments for inference, we used K=10 genetic variants. For each variant, we generated effect allele frequencies $f_{j}∼U(0.1,0.9)$ and allele counts $G_{j}∼\text{Binomial}(2,f_{j})$. We then generated exposure measurements as

$$X=\alpha_{X}+G^{T}\beta_{X}+\gamma_{X}U+\epsilon_{X}$$

and then simulated the outcome and the selection indicator as previously described.

The effects of genetic instruments on the exposure were specified so that approximately 5% of variation in X was explained by G. This was achieved by setting $\beta_{X}=\sqrt{2/19}$ in simulations with a single instrument for inference, and generating the $\beta_{Xj}$ from a $N(0,0.05^{2})$ distribution, left‐truncated at 0.15, in simulations with multiple instruments. The causal effect of interest was assigned values θ=0.2 to facilitate a power comparison between the various methods, or θ=0 to explore the methods' performance under the null causal hypothesis. The intercepts $\alpha_{X},\alpha_{Y}$ were set equal to zero for simplicity, while the confounding effects $\gamma_{X},\gamma_{Y}$ were set equal to 1.

For the selection effects $\beta_{R},\delta_{R}$, we considered three scenarios. In our first scenario, selection was affected by the outcome but not the exposure ($\beta_{R}=0,\delta_{R}=1$). In the second scenario, selection was affected by the exposure but not the outcome ($\beta_{R}=1,\delta_{R}=0$). In the third scenario, both the exposure and the outcome were causes of selection ($\beta_{R}=\delta_{R}=0.5$). In all scenarios, the $R^{2}$ statistic for the effects of X and Y on selection was approximately 20%. The instrument‐selection effect was set equal to $\gamma_{R}=0.5$, corresponding to an $R^{2}$ value of approximately 2% for Z. Finally, the intercept $\alpha_{R}$ was again tuned to ensure that fully‐observed data were available for 50% of individuals.

We considered both one‐sample and two‐sample MR designs. With a single instrument for inference, we used the Wald ratio to estimate the causal effect in both cases. With multiple instruments, we used two‐stage least squares for one‐sample MR and the summary statistics approach for two‐sample MR. The summary statistics approach can also be implemented in one‐sample MR; simulation results from such an implementation are reported in Table S5. 2SLS was implemented using the R package ivreg, with 100 bootstrap iterations used to compute the standard errors for the IVsel methods. IVW was implemented using the R package MendelianRandomization. For Wald ratios and IVW estimates, we used second‐order approximations for the MR standard errors. The sample size used in our simulations was $N=10^{4}$ for one‐sample MR and $N_{1}=N_{2}=10^{4}$ for two‐sample MR. The simulations were replicated $10^{4}$ times for MR with a single instrument for inference, and $10^{3}$ times for MR with multiple instruments, to reduce the computational burden.

#### 4.2.2 Simulation results

Between the value of the causal effect θ, the selection effects $\beta_{R},\delta_{R}$, the number of instruments for inference and the MR study design (one‐sample or two‐sample), our simulation resulted in 24 distinct scenarios. The results are reported in Table 3 for MR simulations with a single instrument for inference, and in Table 4 for simulations with multiple instruments.

**TABLE 3:** Performance of the various selection bias adjustment methods in Mendelian randomization simulations with a single genetic instrument for inference.

	θ=0.2					θ=0			
Method	Causal	Emp SD	StdErr	Cover	Power	Causal	Emp SD	StdErr	Type I
	One‐sample MR–Y→R								
CCA	0.143	0.056	0.059	0.847	0.696	−0.001	0.054	0.054	0.052
IPW	0.143	0.039	0.049	0.847	0.886	−0.001	0.038	0.044	0.023
Heckman	0.199	0.061	0.065	0.962	0.882	−0.002	0.058	0.058	0.053
TTW	0.203	0.343	0.340	0.947	0.094	−0.005	0.304	0.301	0.052
Oracle	0.199	0.044	0.049	0.971	0.986	−0.001	0.044	0.044	0.050
	One‐sample MR–X→R								
CCA	0.266	0.060	0.068	0.866	0.985	−0.001	0.059	0.058	0.051
IPW	0.265	0.059	0.067	0.868	0.986	−0.001	0.058	0.058	0.049
Heckman	0.200	0.045	0.051	0.971	0.985	−0.001	0.044	0.044	0.049
TTW	0.202	0.075	0.073	0.957	0.814	−0.001	0.046	0.046	0.053
Oracle	0.199	0.044	0.049	0.972	0.987	−0.001	0.044	0.044	0.050
	One‐sample MR–X,Y→R								
CCA	0.054	0.070	0.072	0.484	0.112	−0.119	0.068	0.064	0.449
IPW	0.056	0.049	0.057	0.252	0.130	−0.118	0.047	0.049	0.672
Heckman	0.198	0.064	0.071	0.970	0.823	−0.001	0.063	0.063	0.051
TTW	0.182	0.421	0.387	0.970	0.036	−0.029	0.974	0.919	0.004
Oracle	0.199	0.044	0.049	0.974	0.988	−0.001	0.044	0.044	0.049
	Two‐sample MR–Y→R								
CCA	0.144	0.058	0.059	0.841	0.690	0.001	0.053	0.054	0.048
IPW	0.144	0.041	0.049	0.835	0.875	0.000	0.038	0.044	0.024
Heckman	0.200	0.064	0.065	0.950	0.873	0.000	0.058	0.058	0.052
TTW	0.213	0.344	0.340	0.946	0.101	−0.003	0.305	0.301	0.053
Oracle	0.200	0.049	0.049	0.951	0.984	0.000	0.044	0.044	0.054
	Two‐sample MR–X→R								
CCA	0.268	0.068	0.068	0.831	0.980	0.000	0.059	0.058	0.054
IPW	0.268	0.067	0.067	0.830	0.983	0.000	0.059	0.058	0.052
Heckman	0.202	0.051	0.051	0.947	0.981	0.000	0.044	0.044	0.055
TTW	0.204	0.081	0.074	0.950	0.791	0.000	0.046	0.046	0.055
Oracle	0.201	0.050	0.049	0.946	0.984	0.000	0.044	0.044	0.053
	Two‐sample MR–X,Y→R								
CCA	0.058	0.072	0.072	0.483	0.128	−0.117	0.065	0.064	0.439
IPW	0.057	0.051	0.057	0.264	0.149	−0.117	0.046	0.049	0.668
Heckman	0.202	0.071	0.072	0.949	0.806	0.000	0.063	0.063	0.051
TTW	0.260	0.434	0.390	0.957	0.077	0.022	0.342	0.331	0.052
Oracle	0.201	0.050	0.049	0.945	0.983	0.000	0.044	0.044	0.053

**TABLE 4:** Performance of the various selection bias adjustment methods in Mendelian randomization simulations with multiple genetic instruments for inference.

	θ=0.2					θ=0			
Method	Causal	Emp SD	StdErr	Cover	Power	Causal	Emp SD	StdErr	Type I
	One‐sample MR (2SLS)–Y→R								
CCA	0.159	0.053	0.055	0.889	0.823	0.013	0.051	0.052	0.042
IPW	0.152	0.040	0.046	0.872	0.940	0.006	0.037	0.043	0.028
Heckman	0.205	0.059	0.060	0.955	0.938	0.010	0.054	0.056	0.048
TTW	0.226	0.332	0.338	0.959	0.102	0.004	0.299	0.301	0.057
Oracle	0.206	0.044	0.043	0.947	0.998	0.008	0.043	0.043	0.054
	One‐sample MR (2SLS)–X→R								
CCA	0.066	0.084	0.083	0.638	0.126	−0.138	0.084	0.083	0.363
IPW	0.050	0.059	0.063	0.336	0.103	−0.153	0.061	0.064	0.666
Heckman	0.195	0.074	0.071	0.939	0.780	−0.007	0.073	0.071	0.060
TTW	0.130	0.046	0.042	0.588	0.852	0.006	0.034	0.033	0.062
Oracle	0.208	0.042	0.043	0.951	0.999	0.007	0.043	0.043	0.049
	One‐sample MR (2SLS)–X,Y→R								
CCA	0.077	0.066	0.067	0.541	0.203	−0.103	0.065	0.065	0.333
IPW	0.067	0.048	0.054	0.293	0.206	−0.109	0.045	0.051	0.566
Heckman	0.214	0.062	0.062	0.949	0.928	0.010	0.060	0.061	0.050
TTW	0.379	0.219	0.218	0.876	0.425	0.187	0.221	0.198	0.186
Oracle	0.208	0.043	0.043	0.947	0.997	0.007	0.042	0.043	0.047
	Two‐sample MR (Summary Statistics)–Y→R								
CCA	0.141	0.058	0.062	0.853	0.642	0.002	0.053	0.057	0.032
IPW	0.141	0.041	0.050	0.824	0.859	0.002	0.037	0.044	0.022
Heckman	0.196	0.063	0.068	0.965	0.837	0.001	0.058	0.061	0.041
TTW	0.220	0.349	0.368	0.965	0.078	−0.008	0.316	0.321	0.041
Oracle	0.195	0.049	0.052	0.958	0.968	0.001	0.044	0.046	0.037
	Two‐sample MR (Summary Statistics)–X→R								
CCA	0.252	0.065	0.070	0.902	0.962	0.002	0.058	0.061	0.028
IPW	0.254	0.064	0.070	0.898	0.970	0.002	0.058	0.061	0.026
Heckman	0.190	0.049	0.052	0.960	0.965	0.002	0.043	0.046	0.027
TTW	0.107	0.046	0.045	0.420	0.646	0.000	0.033	0.035	0.048
Oracle	0.192	0.048	0.052	0.967	0.972	0.002	0.043	0.046	0.028
	Two‐sample MR (Summary Statistics)–X,Y→R								
CCA	0.052	0.071	0.075	0.500	0.097	−0.111	0.064	0.067	0.381
IPW	0.056	0.051	0.057	0.261	0.139	−0.115	0.045	0.051	0.643
Heckman	0.193	0.073	0.070	0.931	0.783	0.001	0.063	0.063	0.054
TTW	0.129	0.273	0.282	0.956	0.074	0.024	0.241	0.253	0.043
Oracle	0.198	0.051	0.052	0.952	0.978	0.000	0.044	0.046	0.043

In one‐sample MR with only the outcome affecting missingness, the performance of IVsel methods was similar to that in regression analyses. The methods were able to adjust for selection bias at the expense of increased uncertainty around their causal effect estimates, which was not too pronounced for Heckman's method but was larger for TTW. The MAR approaches exhibited bias for θ=0.2 but not for θ=0; a null causal effect means that G and X are no longer upstream of the selection indicator in the causal diagram of Figure 2, hence selection bias is not induced.^37^

When missingness was affected by the exposure but not the outcome, the performance of IVsel methods improved further and the standard errors produced by the methods were not much larger than those of the oracle analysis. Recall that the standard error of the Wald ratio (7) depends primarily on the standard error ${ŝ}_{Y}$ of the G−Y association and only to a lesser extend on the standard error ${ŝ}_{X}$ of the G−X association. When missingness is only a function of X, the IVsel methods will produce an increased standard error ${ŝ}_{X}$ but this will only have a small impact on $\text{s.e.}(\hat{\theta })$.

CCA and IPW were biased in this simulation, at least for θ≠0, but the direction of bias was different to the scenario with missing outcome values. This is again due to the fact that the MR causal effect estimate is a ratio of two parameter estimates, $\hat{\theta }=\frac{{\hat{\beta }}_{Y}}{{\hat{\beta }}_{X}}$. In Section 4.1, CCA and IPW exhibited downward bias. If such bias arises in the numerator of the Wald ratio, it will result in an underestimation of the MR causal effect. On the other hand, downward bias in the denominator will result in overestimation. In the case of θ=0, selection bias did not appear with missing exposure data because all methods were able to estimate $\beta_{Y}=0$ unbiasedly, meaning that any bias in estimating $\beta_{X}$ was of lesser concern.

When missingness was affected by both the exposure and the outcome, TTW estimates deviated slightly from the true causal effect values. This was because of the substantial uncertainty in $\beta_{X}$ estimates obtained from the TTW method, as seen in the previous section. Similar to how weak instrument bias occurs in traditional MR studies, when ${\hat{\beta }}_{X}$ takes a value close to zero, it can result in unstable causal effect estimates. This happened in a few replications of our simulation, due to the high uncertainty of the TTW method, hence giving the impression of bias. Heckman's method was less affected by this phenomenon because of the method's smaller standard errors.

Finally, a comparison of results from one‐sample MR and two‐sample MR simulations suggests little difference between the two settings in terms of the performance of selection bias adjustment methods.

Table 4 reports results from simulations using multiple genetic instruments for inference. A notable difference from simulations with a single instrument for inference was that the performance of the TTW method deteriorated. In two‐sample MR, the method was still able to obtain unbiased estimates of the SNP‐specific G−X and G−Y associations, but the precision of these estimates was quite low and combining these associations using the IVW formula produced biased causal effect estimates. Similar issues arose in the 2SLS implementation of the method. There are similarities here between the suboptimal performance of TTW estimates and MR analyses with measurement error, where G−X and G−Y associations are also estimated with low precision. Methods aiming to adjust MR analyses for measurement error^38^ could potentially be used to improve the performance of the TTW method, but we have not explored this further.

The precision of TTW estimates depends on the sample size and the strength of the instrument for selection, meaning that the method may perform better in MR analyses with larger sample sizes and/or stronger instruments. In addition, a comparison of Tables 3 and 4 suggests that TTW estimates were more accurate when the method only used a single instrument for inference. Therefore, it may be useful to combine genetic instruments into a polygenic risk score, if possible, and then use the TTW method with a single instrument for inference.

Heckman's method did not face the same issue as TTW due to the smaller degree of uncertainty about its estimates. The method produced estimates with little bias in all scenarios and was the preferred method in this simulation. The performance of complete‐case analysis and IPW was similar to that observed in simulations with a single instrument for inference. Of note was the different direction of bias between one‐sample and two‐sample MR when selection was affected only by the exposure. This was due to the method used in each case. In one‐sample MR, we used the 2SLS approach whose default implementation in R discards individuals with missing values from both the first and the second stage, meaning that selection bias will affect both the exposure and outcome models. The pattern of bias for 2SLS was therefore similar to the single‐instrument simulations where selection was affected by both the exposure and outcome, and this also caused bias for θ=0. On the other hand, in two‐sample MR we used IVW to obtain causal effect estimates. This requires summary statistics for the exposure and outcome to be estimated separately, and with no missing data in the sample for the outcome we were able to estimate unbiasedly the G−Y associations. This resulted in a pattern of bias similar to that observed in simulations with a single instrument and selection depending only on X. In addition, bias in the two‐sample case was not observed when θ=0.

Additional simulation scenarios, both for regression and for MR analyses, are considered in Tables S1–S6 and Figures S2, S5 and S6. In these simulations, we vary some aspects of our simulation design and explore how these affect the performance of IVsel methods in regression and MR analyses. In particular, we consider simulations with different proportions of missing data, different strengths of the selection effects (X−R and Y−R), violations of the IV assumptions for selection, model misspecification, MR analyses with different sample sizes, and different values of the MR causal effect.

## 5 APPLICATION: EFFECTS OF BMI ON SMOKING IN ALSPAC

We implemented the selection bias adjustment methods in a real‐data application, using data from the Avon Longitudinal Study of Parents and Children (ALSPAC),^39^, ^40^ a longitudinal population‐based study that recruited pregnant women residing in Avon, UK, with expected delivery dates between 1 April 1991 and 31 December 1992. The study included 15,454 pregnancies resulting in 15,589 foetuses, 14,901 of which were alive at 1 year of age. Ethical approval for the study was obtained from the ALSPAC Ethics and Law Committee and the Local Research Ethics Committees. Informed consent for the use of data collected via questionnaires and clinics was obtained from participants following the recommendations of the ALSPAC Ethics and Law Committee at the time. The study website (http://www.bristol.ac.uk/alspac/researchers/our‐data/) contains details on all data that is available through a fully searchable data dictionary and variable search tool.

Using data collected from the offspring, our objective in this application was to assess the relation between BMI and two smoking traits, namely smoking status (ever vs. never) and the number of cigarettes smoked per day on average.

### 5.1 Data and methods

Access to ALSPAC individual‐level data was obtained under application B3838. We used data from both clinic visits (“Teen Focus 4” stage, age 18) and questionnaires (“It's all about you”, age 20). BMI measurements were recorded during the clinic visit. Outcome data for smoking status and number of cigarettes smoked per day were self‐reported as part of the questionnaire. We also obtained genetic data for the MR analyses, and information on participants' sex and maternal variables (smoking before pregnancy, gestational age and highest educational qualification held), which were used as additional covariates in our analysis.

Missing data were present for all variables. Our aim was to investigate the performance of various methods in adjusting for missingness in the exposure and outcome, hence we restricted our analysis to genotyped individuals with fully recorded maternal covariates. This resulted in a sample size of n=7779 individuals.

We performed Mendelian randomization to study the relationship between BMI and smoking outcomes. MR estimates were obtained as Wald ratios, using a polygenic risk score (PRS) as a single instrument for BMI. To construct the PRS, we obtained a list of genetic variants associated with BMI from a previous GWAS study.^41^ In total, 311 variants were matched to our ALSPAC data. Variants were coded so that the effect alleles had risk‐increasing effect on BMI. The score was then constructed using the SNP‐BMI associations from the BMI GWAS as weights. The PRS‐BMI association was estimated from linear regression. The association of the PRS with smoking status was estimated using logistic regression, while the association with the number of cigarettes smoked per day was estimated using linear regression, as well as Poisson regression.

### 5.2 Instruments for selection

For the IVsel methods, we used four different instruments for selection. The first instrument was non‐genetic. As part of the ALSPAC study, a small‐scale randomized trial was conducted when inviting participants to complete the “It's all about you” questionnaire. Participants were randomized to either receive an online invitation, or to receive a printed version of the questionnaire and have a choice on whether to return it in print or online. Mode of delivery of the questionnaire affected its completion, albeit weakly.^42^ Not all ALSPAC participants were enrolled in the trial; consequently, our analyses using this instrument had to be conducted on a smaller set of $n_{\text{RCT}}=5646$ individuals.

The other three instruments were genetic. A recent study investigated genetic associations with ALSPAC participation,^43^ which can be used to construct a polygenic risk score for participation. In order to obtain a reasonably strong instrument, we used a lenient threshold of $10^{-4}$ for inclusion of genetic variants; this resulted in a risk score comprising 178 variants, after LD pruning. More details on the construction of the PRS for participation are given in the Supplementary Material.

To construct the last two instruments, we considered genetic associations with optional participation components in UK Biobank.^18^ We derived two polygenic risk scores, one for variants associated with completion of the Food Frequency questionnaire (FFQ) and one for the Mental Health Questionnaire (MHQ). These risk scores consisted of 13 and 40 SNPs respectively. Arguably, selection into UK Biobank is different to ALSPAC participation, but the two risk scores were reported to have high genetic correlations with ALSPAC participation 0.488 for FFQ, 0.627 for MHQ,^18^ and the SNPs included in these scores did not associate with BMI or any of the outcomes in our study, hence they constitute valid instruments.

### 5.3 Results

**FIGURE 3:**
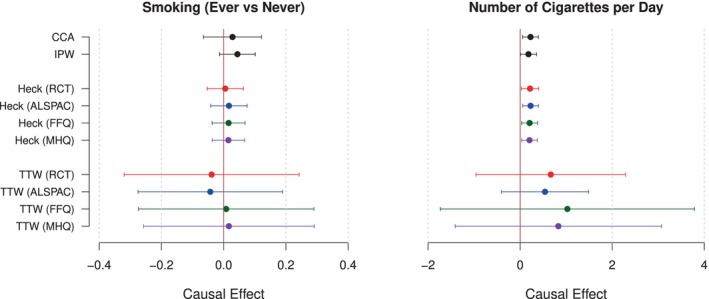
MR estimates of the effect of BMI on smoking status (left) and number of cigarettes smoked per day (right), obtained from ALSPAC data using various methods to adjust for missing data in BMI and smoking outcomes. Colors indicate which instrument for selection is used each time (red: RCT for participation, blue: ALSPAC risk score, green: SNPs associated with FFQ completion in UK Biobank, purple: SNPs associated with MHQ completion in UK Biobank, black: method requires no instrument for selection).

We implemented complete‐case analysis, IPW and the IVsel methods to estimate the effects of BMI on the two smoking outcomes in our study. Results are reported in Figure 3 and in Table S7. Our analysis identified a risk‐increasing effect of genetically elevated BMI on the number of cigarettes smoked per day, with effect estimates of 0.226 (95% CI (0.056,0.396)) by complete‐case analysis and 0.182 (95% CI (0.007,0.356)) by inverse probability weighting. Heckman's method confirmed this result, with all four implementations of the method suggesting similar effect estimates as CCA and IPW. The TTW method suggested a larger effect, but the method's precision was low and the effect did not pass the 5% significance threshold.

For smoking status, the point estimates and confidence intervals plotted in Figure 3 represent log‐odds ratios of increase in odds of smoking per unit increase in BMI. There was little evidence of a causal effect of genetically elevated BMI on smoking status in our analysis. Causal effect estimates were consistent across methods, although the TTW method again produced larger standard errors and wide confidence intervals.

Implementations of the IVsel methods using different instruments for selection produced similar causal effect estimates, which is reassuring. Among the four instruments, the ALSPAC‐derived risk score was fairly strong, while the other three instruments were rather weak and produced F statistics near or below 10 (Table S10). Accordingly, the TTW method attained higher precision when implemented using the ALSPAC risk score, confirming the benefits of using strong instruments when applying the IVsel methods in practice. Results from Heckman's method exhibited less variation with different instruments; for binary outcomes, the method seemed to be less sensitive to instrument strength.

Results obtained using the IVsel approaches were in decent agreement with those obtained from the more traditional methods. Since the IVsel methods make more general assumptions about the missingness mechanism than CCA or IPW, their implementation here is useful as a form of sensitivity analysis, and suggests that selection bias is unlikely to have had a serious impact in our analysis.

Reference 44 used ALSPAC data to identify an effect of BMI on early‐onset smoking (at age 15–16) in female participants, but no effect of BMI on late‐onset smoking (age 17+) in either males or females. In this application we used smoking data from adulthood (age 20+), hence our findings are consistent with the lack of a late‐onset effect in Reference 44. In sex‐stratified analyses, not reported here, we observed no effect of BMI on smoking status in either males males or females. Evidence of a reverse effect of smoking on BMI has been observed in Reference 45, meaning that early‐onset smoking could act as a confounder between BMI and smoking at ages 18–20, which could induce bias in our analysis if BMI affects smoking at an early age. An exhaustive analysis of the relationship between BMI and smoking across different age groups^46^ would be a challenging task, hence we decided to limit our attention to the age groups in our dataset. We should mention, however, that studies using larger datasets, including the UK Biobank, have identified effects of obesity on smoking initiation,^47^, ^48^ so our null result could be due to the small sample size of ALSPAC. This could also explain the seemingly counterintuitive finding that BMI associates with smoking intensity but not smoking status in our analysis: smoking status is a binary variable, hence the MR analysis with smoking status as an outcome has lower power than the analysis with number of cigarettes as an outcome.

## 6 DISCUSSION

In this paper we have discussed how to use instruments for selection in order to adjust for selection bias due to MNAR data, in regression studies and MR analyses with individual‐level data. This is an established approach in econometrics and has recently found applications in biomedical research.^6^, ^49^, ^50^ In this paper, we have argued that this approach can readily be extended to Mendelian randomization studies. We have conducted a simulation study to assess its performance, and utilized it in a real‐data application studying the effects of BMI on smoking traits.

There is an extensive literature on Heckman‐type selection models and it would be difficult to cover it exhaustively. Instead, we have decided to focus on two approaches: Heckman's original method and its extension by Tchetgen Tchetgen and Wirth. Both methods were able to adjust for selection bias in a wide range of simulation scenarios, at the expense of lower precision in parameter estimates. The methods performed best with strong instruments and continuous covariates and outcomes. Potential violations of the instrumental variable assumptions should be carefully considered before using the methods in practice, as such violations can induce substantial biases. Comparing the two methods, Heckman's approach usually produced smaller standard errors; however, Heckman's normality assumptions mean that the TTW approach is more widely applicable, as illustrated in simulations with binary and discrete outcomes.

Our simulation study explored a wide range of scenarios and we hope it can provide guidelines to applied researchers about the performance of the IVsel methods in both traditional regression analyses and MR. Nevertheless it has not been possible to cover every simulation scenario of interest. For example, we did not vary the sample size and the number of genetic instruments for inference. A large sample size will increase the computational cost of running the IVsel methods but will also improve the methods' performance; on the other hand, a smaller sample size will further increase the uncertainty around causal effect estimates, which can affect TTW particularly. A large number of instruments for inference could be particularly worrying for the 2SLS approach because it will require solving a high‐dimensional optimization problem to estimate the G−X associations. We have also not considered simulations with interactions between variables in the selection model; such interactions are known to have a significant impact on the magnitude of selection bias.^51^, ^52^

Our work admits a number of methodological extensions. For example, it may be possible to formulate our IVsel‐adapted 2SLS procedure as a structural equation model, which would improve its computational efficiency by facilitating estimation of standard errors and avoid the need to use a bootstrap algorithm. Another potential extension would be to use the sample selection framework in order to compute bounds for the MR causal effect under missing data. Such bounds have already been proposed for regression‐based analyses, both in Tchetgen Tchetgen and Wirth's paper and elsewhere.^53^, ^54^, ^55^ The bounds reported in these papers can in principle be extended to MR; for example, when estimating a Wald ratio, one can compute bounds for the instrument‐exposure and instrument‐outcome associations separately, and combine them to derive worst‐case bounds for the causal effect of interest. Note, however, that these bounds can only be computed for binary (or at least bounded) variables. In addition, the bounds can be very wide; for example, if the bounds for both the instrument‐exposure and the instrument‐outcome effect include 0, the range of possible values for the causal effect of interest will span the entire real line. Another interesting extension of our work could be to combine the IVsel approaches with methods that offer robustness to weak instruments. Such methods may include limited‐information maximum likelihood for point estimation and Anderson‐Rubin or conditional likelihood ratio tests for hypothesis testing. Using these approaches in conjunction with the IVsel methods may improve the methods' performance, especially for the TTW method. In addition, it is worth noting that Heckman‐type selection models are not the only approach for addressing selection bias due to MNAR data. For example, some authors have explored the use of shadow variables instead of instruments for selection.^56^ Another approach is to use multiple imputation, for example through the NARFCS algorithm.^57^ This approach has already proved useful in traditional epidemiologic studies, but to the best of our knowledge it has not been implemented in MR studies. Some attempts to combine imputation with Heckman‐type selection models have also been made in the literature.^58^ On the other hand, the literature on two‐sample summary‐data MR has also given rise to selection bias adjustment methods, designed specifically for studies of disease progression. Such methods include^59^ index event bias adjustment and SlopeHunter.^60^ A comparison between IVsel and these methods is beyond the scope of the current paper, but would be an interesting direction of future research.

Some R code to implement the IVsel methods is available in the GitHub page https://github.com/agkatzionis/IVsel. We hope that our code and results will prove useful to applied researchers looking to address issues related to selection bias and missing data in their work.

## CONFLICT OF INTEREST STATEMENT

The authors declare no potential conflict of interests.

## Supporting information




**Data S1:** Supporting Information.

## Data Availability

The code used to implement the methods described in our manuscript and to generate data for our simulation study is freely available in the GitHub repository: https://github.com/agkatzionis/IVsel. The datasets used for the real‐data application were obtained from the Avon Longitudinal Study of Parents and Children (ALSPAC). Restrictions apply to the availability of these data, which were used under license (application B3838) for this study. Access to the ALSPAC data can be obtained upon request via the website: https://www.bristol.ac.uk/alspac/.
