# Supplementary material for: Using instruments for selection to adjust for selection bias in Mendelian randomization
Source: Stat Med. 2024 Jul 22;43(22):4250–71. doi: 10.1002/sim.10173 (PMC13022821; doi:10.1002/sim.10173)
Supplement: Supplementary file 1 — Data S1: Supporting Information. [file SIM-43-4250-s001.zip › Using Instruments for Selection in MR (Supplement) - StatsInMed - Submission 3.pdf]

Received: Added at production | Revised: Added at production | Accepted: Added at production

DOI: xxx/xxxx

ARTICLE TYPE

# Using Instruments for Selection to Adjust for Selection Bias in Mendelian Randomization

Apostolos Gkatzionis*[1,2] | Eric J. Tchetgen Tchetgen[3] | Jon Heron[1,2] | Kate Northstone[2] | Kate Tilling[1,2]

[1]MRC Integrative Epidemiology Unit, University of Bristol, Bristol, United Kingdom
[2]Population Health Sciences, Bristol Medical School, University of Bristol, Bristol, United Kingdom
[3]Department of Statistics and Data Science, Wharton School, University of Pennsylvania, Pennsylvania, USA

**Correspondence**
*Corresponding author. Email: apostolos.gkatzionis@bristol.ac.uk

**Present Address**
MRC Integrative Epidemiology Unit, University of Bristol, Oakfield House, Oakfield Grove, Bristol, BS8 2BN, United Kingdom

Selection bias is a common concern in epidemiologic studies. In the literature, selection bias is often viewed as a missing data problem. Popular approaches to adjust for bias due to missing data, such as inverse probability weighting, rely on the assumption that data are missing at random and can yield biased results if this assumption is violated. In observational studies with outcome data missing not at random, Heckman's sample selection model can be used to adjust for bias due to missing data. In this paper, we review Heckman's method and a similar approach proposed by Tchetgen Tchetgen and Wirth (2017). We then discuss how to apply these methods to Mendelian randomization analyses using individual-level data, with missing data for either the exposure or outcome or both. We explore whether genetic variants associated with participation can be used as instruments for selection. We then describe how to obtain missingness-adjusted Wald ratio, two-stage least squares and inverse variance weighted estimates. The two methods are evaluated and compared in simulations, with results suggesting that they can both mitigate selection bias but may yield parameter estimates with large standard errors in some settings. In an illustrative real-data application, we investigate the effects of body mass index on smoking using data from the Avon Longitudinal Study of Parents and Children.

**KEYWORDS:**
Mendelian randomization, instrumental variables, selection bias, Missing Not At Random, Heckman selection model, ALSPAC

## SUPPLEMENTARY MATERIAL

This part contains the supplementary material for the paper "Using Instruments for Selection to Adjust for Selection Bias in Mendelian Randomization". It includes a brief summary of the TTW method for logistic and Poisson regression, additional simulation results, as well as more details about our real-data application.

## RELAXING HECKMAN'S MODELLING ASSUMPTIONS

An important limitation of Heckman's sample selection model is that it requires a normally distributed outcome and a probit model for the selection process. Various extensions or modifications of Heckman's modelling assumptions have been proposed in

the literature. Parametric extensions include a probit model for binary outcomes[1], a Poisson distribution for discrete outcomes[2] and a $t$ distribution for continuous outcomes[3] among others. Many of these extensions relied on maximum likelihood estimation, instead of Heckman's original two-stage algorithm, which is harder to extend to non-normal settings.

In terms of non-parametric extensions,[4] and[5] used Heckman's two-stage approach but replaced the normal regression and probit selection models with semi- or non-parametric equivalents.[6] combined Heckman's probit selection model with a non-parametric model for the outcome, $Y = \mu(X) + \epsilon$, and used splines to model the regression function $\mu(X)$. One of the main challenges these non-parametric approaches faced was to establish parameter identification. This is not a concern in many parametric models, including Heckman's original setting, because the restrictive nature of a parametric model is often sufficient to guarantee identification. However, it can be a hard task in non-parametric settings; for example, Das et al. established identification for their basis function expansion algorithm only up to an additive constant.

As discussed in our manuscript,[7] proposed general-purposed general-purpose identification condition and developed a maximum likelihood estimation algorithm that can be applied to a wide range of parametric models. We presented the algorithm for linear regression in the main part of our manuscript, and refer to the next Section of this Supplement for an overview of logistic and Poisson regression. The appendix of Tchetgen Tchetgen and Wirth's paper also contains a comparison of their work with that of[6]. In related work,[8] proposed an alternative identification condition, which can be easier to verify than the homogeneous selection bias assumption, along with a different estimation strategy.

Finally, some authors have considered using the sample selection framework in order to compute bounds for the MR causal effect under missing data[9,10,11].

# TTW FOR LOGISTIC AND POISSON REGRESSION

In this section, we outline the TTW method for binary and discrete outcomes. We focus on stating the homogeneous selection bias assumption and outlining the estimation process, and refer to[7] for more details.

## Logistic Regression

Suppose that the outcome $Y$ is a binary variable, and that we wish to model it using logistic regression. In this case, our interest lies in estimating the log-odds ratio

$$\text{logit}\mathbb{P}(Y = 1|X) = \log \frac{\mathbb{P}(Y = 1|X)}{\mathbb{P}(Y = 0|X)} = X^T \beta$$

Selection bias in the odds ratio scale can be quantified as the log-odds ratio for $Y = 1$ between individuals with observed and unobserved outcome values:

$$\log \frac{\mathbb{P}(Y = 1|X, Z, R = 1)/\mathbb{P}(Y = 0|X, Z, R = 1)}{\mathbb{P}(Y = 1|X, Z, R = 0)/\mathbb{P}(Y = 0|X, Z, R = 0} = \omega(X, Z) \tag{1}$$

The homogeneous selection bias assumption states that selection bias must be independent of the instrument on a log-odds ratio scale, so $\omega(X, Z)$ must be a function of $X$ only and cannot depend on $Z$. As with linear regression, this assumption is not strictly necessary for identification in logistic regression models but can be used to establish identification in more general models where the goal is to make inference on the regression function $\mu(X; beta) = \text{logit}\mathbb{P}(Y|X)$.

Under the homogeneous selection bias assumption, the full-data regression parameter $\beta$ of a logistic regression model can be estimated using observed data via the relation

$$\text{logit}\mathbb{P}(Y = 1|X, Z, R = 1) = X^T \beta + \omega(X) - \log\left(e^{\omega(X)} \lambda(X, Z) + 1 - \lambda(X, Z)\right) \tag{2}$$

where $\lambda(X, Z) = \mathbb{P}(R = 1|X, Z, Y = 0)$ satisfies

$$\pi(X, Z) = \frac{1}{1 + e^{X^T \beta}} \lambda(X, Z) + \frac{e^{X^T \beta}}{1 + e^{X^T \beta}} \frac{1}{1 + (1 - \lambda(X, Z))e^{-\omega(X)}/\lambda(X, Z)} \tag{3}$$

Equations (2)-(3) can be used to construct a likelihood for the observed-data regression and the propensity score respectively. Subject to parametric assumptions for $\omega(X)$ and $\lambda(X, Z)$, the likelihood can then be optimized to estimate the regression coefficient $\beta$, similar to the linear regression case that we described in the main part of our manuscript. One difference is that, because

the selection function and linear predictor term are included both in (2) and in (3), it is not possible to split the maximum likelihood procedure in two parts and implement partial-likelihood optimization, therefore full optimization over all parameters simultaneously is the only option.

## Poisson Regression

If the outcome $Y$ represents count data, a common modelling assumption is to assume that they come from a Poisson distribution. Written as a generalized linear model, Poisson regression admits the logarithmic link function:

$$\mathbb{E}(Y|X) = \exp\left\{X^T\beta\right\}$$

The homogeneous selection bias assumption again functions on the same scale as the regression coefficient of interest. Selection bias can be quantified as the ratio of outcome values between individuals with observed and unobserved outcomes,

$$\frac{\mathbb{E}(Y|X,Z,R=1)}{\mathbb{E}(Y|X,Z,R=0)} = \nu(X,Z) \tag{4}$$

and the homogeneous selection bias assumption states that $\nu(X,Z)$ must be a function of $X$ but not $Z$. Similar to linear and logistic regression, the assumption can be used to obtain a relationship between the full-data regression coefficient $\beta$ and the observed-data regression,

$$\log\mathbb{E}(Y|X,Z,R=1) = X^T\beta + \log\nu(X) - \log\left(\nu(X)\pi(X,Z) + 1 - \pi(X,Z)\right) \tag{5}$$

This formula facilitates a maximum likelihood estimation process for $\beta$ that only requires access to the observed data, along with parametric assumptions for the selection function $\nu(X)$ and the propensity score $\pi(X,Z)$. The same homogeneity assumption and maximum likelihood estimation procedure can be used for extensions of the standard Poisson regression model where $\mu(X) = \log\mathbb{E}(Y|X)$ is not assumed to have a linear form.

## Assessing the Homogeneous Selection Bias Assumption

The homogeneous selection bias assumption offers a general, unifying framework for establishing parameter identification for the TTW method in a wide range of statistical models. However, the assumption can be difficult to verify in practice, since the selection function ($\delta$, $\omega$ or $\nu$) will depend on both observed and unobserved outcome values.

In their work, Tchetgen Tchetgen and Wirth identified a class of statistical models where the homogeneity assumption can be shown to hold. This class contains models in which the outcome $Y$ does not directly influence the selection indicator $R$, but instead there are common causes $U$ that influence both the outcome and selection, and the instrument $Z$ does not interact with $U$ in the selection model. The causal diagram in Figure S3 provides an illustration. The homogeneous selection bias assumption is more likely to hold if effect modifiers for the $Z-R$ association are included as covariates in the $X-Y$ regression, which is hence recommended by the authors.

In applications not characterized by Figure S3, intuitive arguments can be made about the validity of the homogeneous selection bias assumption. For example, when using questionnaire data, it has been suggested to use interviewer characteristics as an instrumental variable. Such interviewer characteristics can affect an individual's likelihood of responding to questions, but any effect they may have on the overall magnitude of selection bias is likely to be small, so the homogeneity assumption can be claimed to hold approximately.

We mention that[8] proposed an alternative identification and modelling strategy for non-parametric estimation of statistical models with outcome data missing not at random. Their identification assumption can be easier to verify in practice, while estimation relies on extensions of established techniques such as IPW and outcome regression. The scope of that paper is hence similar to the TTW method presented here, but we have not explored it further and refer to the paper for more details.

# ADDITIONAL SIMULATION RESULTS

We now present results from additional simulations that complement those reported in the paper. As in the main part of our paper, we first discuss simulations for regression analyses and then consider simulations for MR studies.

## Regression Analyses

In the regression setting, we conducted six additional sets of simulations. First, we replicated the simulations of Table 1 with a null association between the covariate and the outcome ($\beta = 0$). Second, we considered different values for the selection effect parameters ($\alpha_R, \beta_R, \gamma_R, \delta_R$). Third, we induced $Z - X$ and $Z - Y$ associations of varying strength and studied how these affect the simulation results. Fourth, we briefly considered a weak-instrument simulation where the instrument was also binary. Fifth, we considered a simulation scenario in which the outcome $Y$ is not a cause of selection $R$, but the two variables are confounded instead. And sixth, we conducted simulations in which the outcome was continuous but not normally distributed, to asses how this form of model misspecification affects the performance of the various methods.

## Null $X - Y$ Association

First, we provide simulation results for the scenarios considered in Table 1, but implemented for a null value of the parameter of interest. We ran these simulations by setting $\beta = 0$ instead of $\beta = 0.1$, and keeping all other aspects of the simulation design unchanged. In Table S1 below, we report average parameter estimates, their empirical standard deviation, model-based standard errors and the Type I error rate of 95% confidence intervals for these simulations.

TABLE S1 HERE

The pattern of selection bias of this simulation was very similar to what we observed for $\beta = 0.1$. This suggests that the true value of the $X - Y$ association has little impact on the magnitude of selection bias in regression analyses. The IVsel methods produced unbiased point estimates and maintained nominal Type I error rates in all simulation scenarios except in the presence of pleiotropy ($Z \to Y$). Of note was the fact that Heckman's method exhibited a reasonable performance in the simulation with a binary outcome.

## Selection Effects

In our next simulation experiment, we varied the values of the selection effect parameters $\alpha_R, \beta_R, \gamma_R, \delta_R$. Recall that $\alpha_R$ represents the intercept in a logistic regression model for the selection indicator $R$, while $\beta_R, \gamma_R, \delta_R$ represent the effects of the covariate, instrument and outcome on the selection indicator respectively. Since $\gamma_R$ represents instrument strength, we have already explored the performance of IVsel methods for various values of that parameter (Table 2), but the results are presented again here for completeness.

We varied the values of these four parameters one at a time, starting from the baseline simulation scenario in which $\alpha_R = -0.5$, $\beta_R = 0.5$, $\gamma_R = 0.4$, $\delta_R = 0.5$. The intercept $\alpha_R$ determines the proportion of individuals for which the outcome is observed; in our baseline simulation, that proportion was 50%. In the new simulations, we used $\alpha_R$ values that resulted in 10%, 20%, 35%, 65%, 80% and 90% of individuals having observed outcomes. For the effects of the covariate ($\beta_R$) and outcome ($\delta_R$) on selection, we experimented with the values 0, 0.1, 0.2, 0.5, 1, 1.5. Note that the simulations with $\beta_R = 0$ or $\delta_R = 0$ correspond to scenarios where only one of $X, Y$ affects selection, similar to those reported in Table 1. Finally, for the instrument strength parameter $\gamma_R$, we used the values 0.08, 0.2, 0.27, 0.4, 0.6 and 0.95 which corresponded approximately to $R^2$ statistic values of 0.1%, 0.5%, 1%, 2%, 5% and 10% respectively, as previously discussed. All simulations were conducted for a target parameter of $\beta = 0.1$. For ease of presentation, the results are reported in graphical form in Figure S1.

The IVsel methods remained unbiased regardless of the proportion of individuals for which the outcome was observed, while for IPW and complete case analysis, selection bias was stronger when 50% of individuals were selected. This value results in the strongest $X - Y$ interaction in a log-probability model for selection, which is known to increase the effects of selection bias[12,13]. The standard errors of all methods were larger for smaller values of $\alpha_R$, due to the fact that the $X - Y$ association parameter had to be estimated in a smaller sample. This was particularly problematic for the TTW method, whose standard errors were too wide to be of practical use when the outcome was observed in only 10% or 20% of individuals.

Differences in the performance of the various methods were observed when we varied the strength of the $X - R$ association. The MAR-based methods exhibited small bias for small values of $\beta_R$. Larger values of $\beta_R$ meant stronger selection effects and

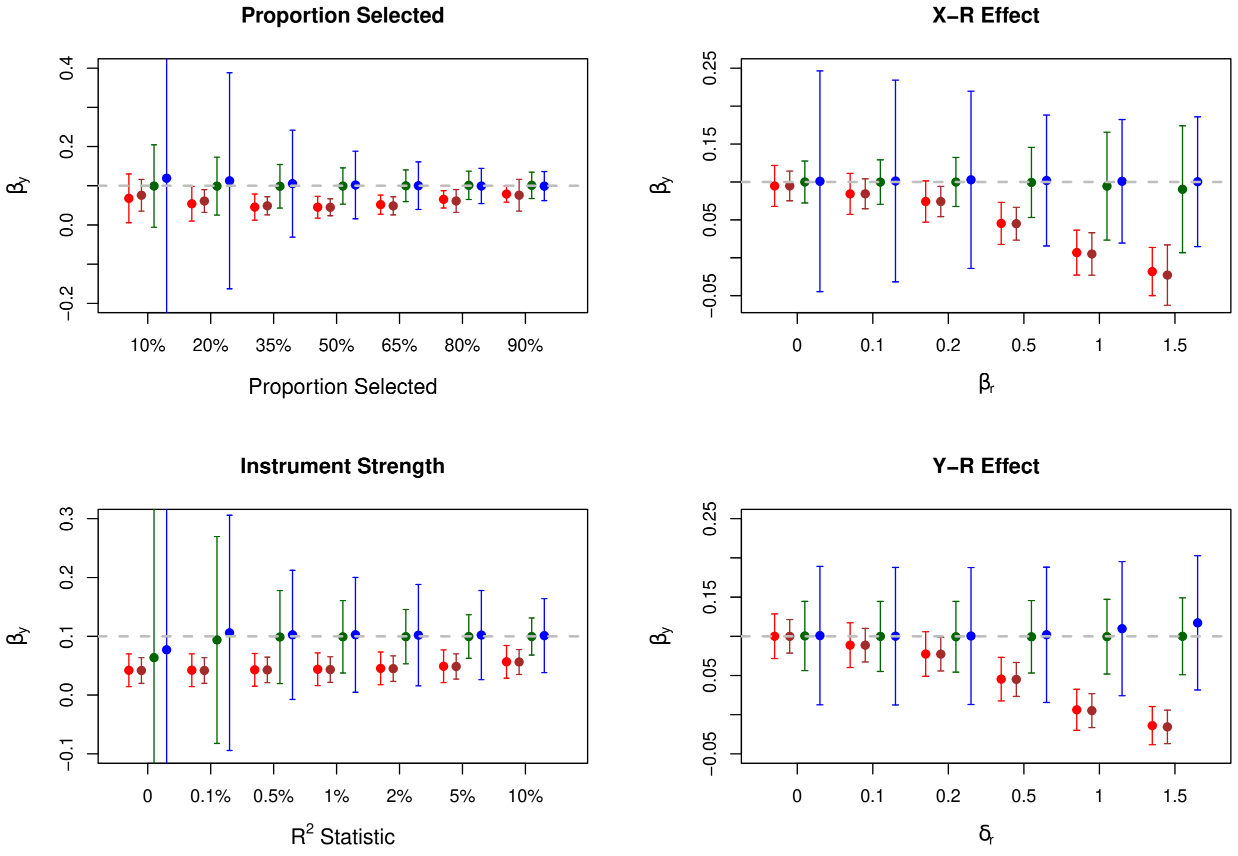


**FIGURE S1** Performance of the various selection bias adjustment methods in regression analyses, for different values of the selection effect parameters $\alpha_R$ (top left), $\beta_R$ (top right), $\gamma_R$ (bottom left) and $\delta_R$ (bottom right).

bigger differences between individuals for which the outcome is observed compared to those for which the outcome is unobserved, so the impact of selection bias on complete-case and IPW estimates increased. Heckman's method remained unbiased for strong $X - R$ associations, but exhibited increased standard errors. Surprisingly, the opposite pattern was observed for the TTW method, whose performance improved for large $\beta_R$ values. We speculate that this may be due to the fact that a stronger $X - R$ association means the method has more information with which to model the selection process.

As discussed in the main part of our manuscript, the instrument strength parameter $\gamma_R$ affects the precision of IVsel estimates. The IVsel methods remain unbiased and maintain nominal coverage across $\gamma_R$ values, but the uncertainty about their parameter estimates increases substantially for weak instruments, at least when the methods are implemented for continuous outcomes. Figure S1 confirms these observations. The performance of IPW and complete-case analysis is barely affected by $\gamma_R$, since these two methods did not utilize the instrument $Z$ (the slight differences observed in the performance of CCA and IPW are because modifying the proportion of variation in selection that is explained by the instrument $Z$ will also modify the proportion of variation explained by $X$ and $Y$ and slightly alter the performance of these methods).

Finally, we performed simulations with different values of the $Y - R$ selection effect. The two MAR methods were unbiased for $\delta_R = 0$, since this simulation corresponds to a MAR setting, and exhibited increasing degrees of bias for larger values of $\delta_R$. The performance of IVsel methods was less affected by the value of $\delta_R$, although a slight overestimation of the regression parameter was observed for the TTW method for large $\delta_R$ values.

## Z-X and Z-Y Effects

In the main part of our manuscript, we conducted two simulations where we added a direct effect of the instrument for selection $Z$ on the exposure or outcome, and investigated how it affects the performance of the IVsel methods. Our conclusion was that

an instrument-exposure effect is not problematic, but an instrument-outcome effect violates the second instrumental variable assumption for $Z$ and induces bias.

As an extension of these simulations, in this subsection we varied the strength of the $Z - X$ and $Z - Y$ effects. For $Z - X$ effects, we modified the baseline simulation design by generating exposure values as $X = \beta_X Z + \epsilon_X$, $\epsilon_X \sim N(0, 1)$. The values we used for $\beta_X$ were $0, 0.1, 0.2, 0.3, 0.5, 1$, with $\beta_X = 0$ corresponding to the baseline simulation from the main part of our paper. For $Z - Y$ effects, we aimed to explore the magnitude of bias induced under mild violation of the second instrumental variable assumption (a weak-to-moderate instrument-outcome effect). We therefore generated outcome values as $Y = \alpha + \beta X + \gamma Z + \epsilon$, letting the parameter $\gamma$ take values $0, 0.1, 0.2, 0.3, 0.5, 1$ (again, $\gamma = 0$ corresponds to our baseline simulation). Other aspects of the simulation design were left unchanged.

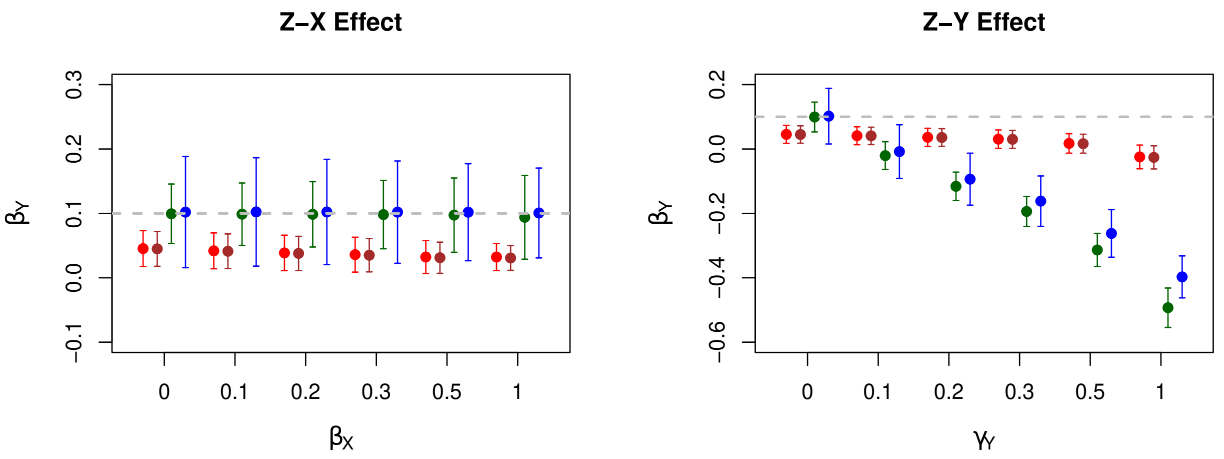


**FIGURE S2** Performance of the various selection bias adjustment methods in regression analyses with an added instrument-exposure (left) or instrument-outcome effect (right).

The results of this set of simulations are reported in Figure S2. They confirm that the IVsel methods are robust to the presence of instrument-exposure effects; for all values of the parameter $\beta_X$, the methods gave similar results. On the other hand, even mild violations of the second instrumental variable assumption were enough to cause bias. Even for the relatively small value of $\gamma = 0.1$, point estimates from Heckman's method and TTW were further away from the true value on average than complete-case and IPW estimates. This suggests that care must be taken when selecting the instrument $Z$ to ensure it is independent of the outcome conditional on exposure.

## Weak Binary Instruments

In our next set of simulations, we explored whether the impact of weak instrument bias changes when using a binary instrument, instead of a continuous one. We did so by considering a single simulation scenario, similar to the "binary instrument" scenario we considered in the main part of the paper, but with the instrument strength parameter set to $\gamma_R = 0.23$, resulting in a simulation where the instrument explained approximately 0.2% of variation in the selection process. This simulation was implemented for either a null ($\beta = 0$) or a positive ($\beta = 0.1$) parameter value. Other aspects of the simulation design were left unchanged.

TABLE S2 HERE

Table S2 reports the results of this simulation experiment. Once again, IVsel methods remained unbiased despite the low instrument strength. Comparing these results with the binary-instrument simulations in Table S1 and in the main part of the

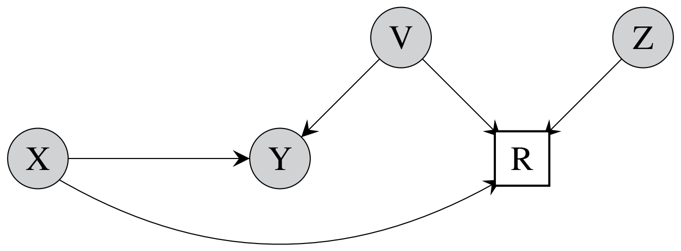


**FIGURE S3** A directed acyclic graph depicting the scenario where the outcome does not cause selection, but a confounder $V$ affects both the outcome and selection.

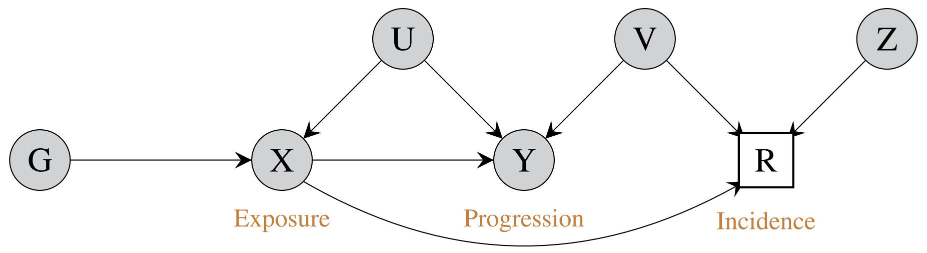


**FIGURE S4** A plausible causal diagram for a Mendelian randomization study of disease progression.

paper, we can also confirm that the precision of IVsel estimates decreased due to low instrument strength. These observations are similar to those obtained when exploring the performance of IVsel methods with weak continuous instruments; there was little change in our results when working with a binary instrument instead of a continuous one.

## Outcome-Selection Confounding

In our third set of simulations, we considered a scenario in which the outcome $Y$ is not a direct cause of the selection indicator $R$, but instead there is an unobserved confounding variable $V$ that affects both the outcome and the selection indicator. This scenario is depicted in the directed acyclic graph of Figure S3.

There are two reasons why this scenario is interesting. First, it was considered in the appendix of Tchetgen Tchetgen and Wirth's paper as a case where the homogeneous selection bias assumption is more likely to hold. The authors prove that the homogeneity assumption will be satisfied if $V$ is a continuous variable, $V$ does not interact with $Z$ in the selection model, and the outcome $Y$ is independent of $R$ and $Z$ conditional on $V, X$. We refer to the paper for a more formal statement of this result.

The second reason why we were interested in outcome-selection confounding is because this setting may be relevant for studies of disease progression. It is well known that such studies are affected by selection bias because disease progression phenotypes can only be observed on individuals who have a disease. This is equivalent to conditioning on disease incidence, which induces collider bias between causes of incidence. Figure S4 illustrates this point for a Mendelian randomization study of disease progression. The outcome of the study is the disease progression trait, while participation is conditional on disease incidence. In such studies it is often implausible to claim that disease progression causes disease incidence. However, there may be unobserved confounders of incidence and progression. Conditional on incidence, such confounders will become associated with the exposure $X$ and result in collider bias.

We performed a simulation to assess whether the pattern of bias is different in the presence of confounding compared to a direct effect of the outcome on the selection coefficient. Our baseline simulation scenario was modified to include the confounder

$V$, resulting in the following simulation design:

$$\begin{aligned} Z, X, V, \epsilon &\sim N(0,1) \\ Y &= \alpha + \beta X + \lambda_Y V + \epsilon \\ R &\sim \text{Logistic}(\pi_R) \\ \text{logit}\left(\pi_R\right) &= \alpha_R + \beta_R X + \gamma_R Z + \lambda_R V \end{aligned}$$

where $\lambda_Y, \lambda_R$ represent the confounder effects on $Y, R$ respectively. We set $\lambda_Y = \lambda_R = 0.5$ and implemented the simulation for either $\beta = 0.1$ or $\beta = 0$. The other simulation parameters were assigned the same values as in our baseline scenario.

TABLE S3 HERE

The results are presented in Table S3. A comparison with our previous results suggests that the effects of selection bias were similar in the two cases. The IVsel methods returned unbiased estimates and confidence intervals with nominal coverage. Bias was still observed for complete-case analysis and IPW, although the bias was somewhat smaller in this case compared to simulations with a direct $Y - R$ effect. This was because the $Y - R$ selection effect was weaker in this simulation, as it was due to confounding and not a direct effect. These results suggest that IVsel methods can be used to account for selection bias in studies of disease progression.

We acknowledge, however, that in practice it can be difficult to identify strong instruments for disease incidence that do not directly affect progression, as would be needed to apply IVsel methods in disease progression studies.

## Outcome Model Misspecification

In our final set of simulations for regression analyses, we explored the performance of the selection bias adjustment methods under model misspecification, and in particular when the outcome is continuous but not normally distributed. We did so by altering the distribution of the error term $\epsilon$ in our model specification. Three distributions were considered:

1. A $t$ distribution, with four degrees of freedom.
2. A log-normal distribution with parameters $\mu_{ln} = 0$ and $\sigma^2_{ln} = \frac{1+\sqrt{5}}{2}$, the latter specified so that the variance of the distribution was equal to 1.
3. A mixture of two normal distributions $N(-2, 0.5^2)$ and $N(2, 0.5^2)$ with mixing proportions 0.25 and 0.75.

These distributions represent three different forms of misspecification for the outcome. The $t(4)$ distribution is symmetric but has heavier tails than the normal distribution, he log-normal distribution is skewed to the right and the mixture distribution is bimodal. Note that we did not violate the linearity of the outcome model in these simulations, we simply misspecified the error terms.

TABLE S4 HERE

We implemented the various selection bias adjustment methods with a normally distributed outcome model. The results are reported in Table S4 and can be compared with the baseline simulation in Table 1. Heckman's method was the least affected by misspecifying the error term in this simulation, and produced practically unbiased parameter estimates. The TTW method was subject to a small degree of bias, while for the CCA and IPW methods, the bias was slightly larger than in our baseline simulation. These findings were observed consistently across the three distributional assumptions for the outcome.

## Mendelian Randomization

We now report the results of additional simulations conducted in the Mendelian randomization setting, to complement the results reported in the paper. In particular, we consider four sets of simulations. First, we report results for one-sample MR simulations with multiple instruments for inference, obtained by computing the selection-adjusted summary statistics. Second, we focus on one-sample MR with a single instrument for inference and vary other aspects of the MR simulation design to explore how they affect the IVsel methods. Third, we explore the power of IVsel methods to detect a causal effect. And fourth, we investigate how the various selection bias adjustment methods are affected by misspecification of the first-stage regression model.

## Summary Statistics for One-sample MR

In the main part of our paper, we discussed two approaches for one-sample MR with multiple instruments for inference. The first approach was based on a modification of the 2SLS method. A second approach was to use the IVsel methods for each genetic instrument for inference separately, compute summary statistics adjusted for selection bias and combine them using the IVW formula. This approach was used in our two-sample MR simulations in the main part of our paper, but there is nothing preventing us from utilizing it in a one-sample setting apart from the fact that using the IVW formula in one-sample MR can induce bias due to sample overlap. This bias is only likely to appear in weak instrument settings though, and at the same time the summary statistics approach is computationally easier to implement. Hence, it may be preferable in simulations with strong instruments where bias due to sample overlap is less likely.

We decided to explore the performance of the summary statistics approach in one-sample MR simulations. For that purpose, we used the same data as those to which we fitted the 2SLS method (see Table 4). For comparison, we implemented a summary-statistics approach not only for the IVsel methods but also for complete-case analysis and IPW.

TABLE S5 HERE

Table S5 contains the results of this simulation experiment. A comparison with the results obtained from the 2SLS method suggests that the impact of sample overlap was rather small. This is not unexpected, since the effect of sample overlap usually manifests as an increase in weak instrument bias. Our MR simulations were parametrized to avoid weak instrument bias and the corresponding $F$ statistics were much larger than the threshold of 10 that is commonly used to detect weak instruments, therefore the impact of weak instrument bias was small. We also note that the performance of the TTW method did not deteriorate further compared to the 2SLS implementation of the method. The only notable difference between 2SLS and the summary statistics approach occurred for the complete-case analysis and IPW methods when selection was a function of the exposure. In this case, selection bias acted in opposite directions for the summary statistics approach compared to the 2SLS method, as we discussed in the main part of our manuscript.

## Additional MR Simulations

In the case of regression analyses, we have covered a wide range of simulation scenarios, varying the values of several simulation parameters. Some conclusions about the performance of IVsel methods in MR settings can be extracted from these regression-based simulations, as we have already mentioned. Here, we explored some of these scenarios explicitly in an MR setting. Specifically, we performed four new sets of simulations. First, we varied the proportion of individuals in the sample with missing data. Second, we conducted simulations with different sample sizes to assess whether the IVsel methods can be used in small-sample MR analyses. And third, we considered violations of the instrumental variable assumptions for selection by inducing direct effects of the instrument for selection on the MR exposure or outcome.

To reduce the number of simulations conducted, we focused on one-sample MR analyses with a single instrument for inference (which could be a polygenic risk score). We also assumed that both the exposure and the outcome affect selection into the MR study, and that both are missing for individuals with missing data. Therefore, the simulations included in this section can be seen as extensions of (and thus compared to) the third set of results in Table 3.

The simulations of this section were generated as follows. For the proportion of missingness, we varied the parameter $\alpha_R$ in the selection model, letting it take values that correspond to 10%, 20%, 35%, 50%, 65%, 80%, 90% of individuals having fully

observed data. For simulations with different sample sizes, we used the following values: $N = 10^3, 2\times10^3, 5\times10^3, 10^4, 2\times10^4, 5\times10^4, 10^5$; simulations with different sample sizes were only repeated $10^3$ times for each $N$ value to reduce computational cost. For simulations with a $Z-X$ effect, we modified the generation of exposure data to $X = \alpha_X + \beta_X G + \gamma_X U + \lambda_X Z + \epsilon_X$, using the values $\lambda_X = 0, 0.1, 0.2, 0.3, 0.4, 0.5$ for the instrument-exposure parameter. For a $Z-Y$ effect, we generated outcome data from $Y = \alpha_Y + \theta X + \gamma_Y U + \lambda_Y Z + \epsilon_Y$, using $\lambda_Y = 0, 0.1, 0.2, 0.3, 0.4, 0.5$ as possible values.

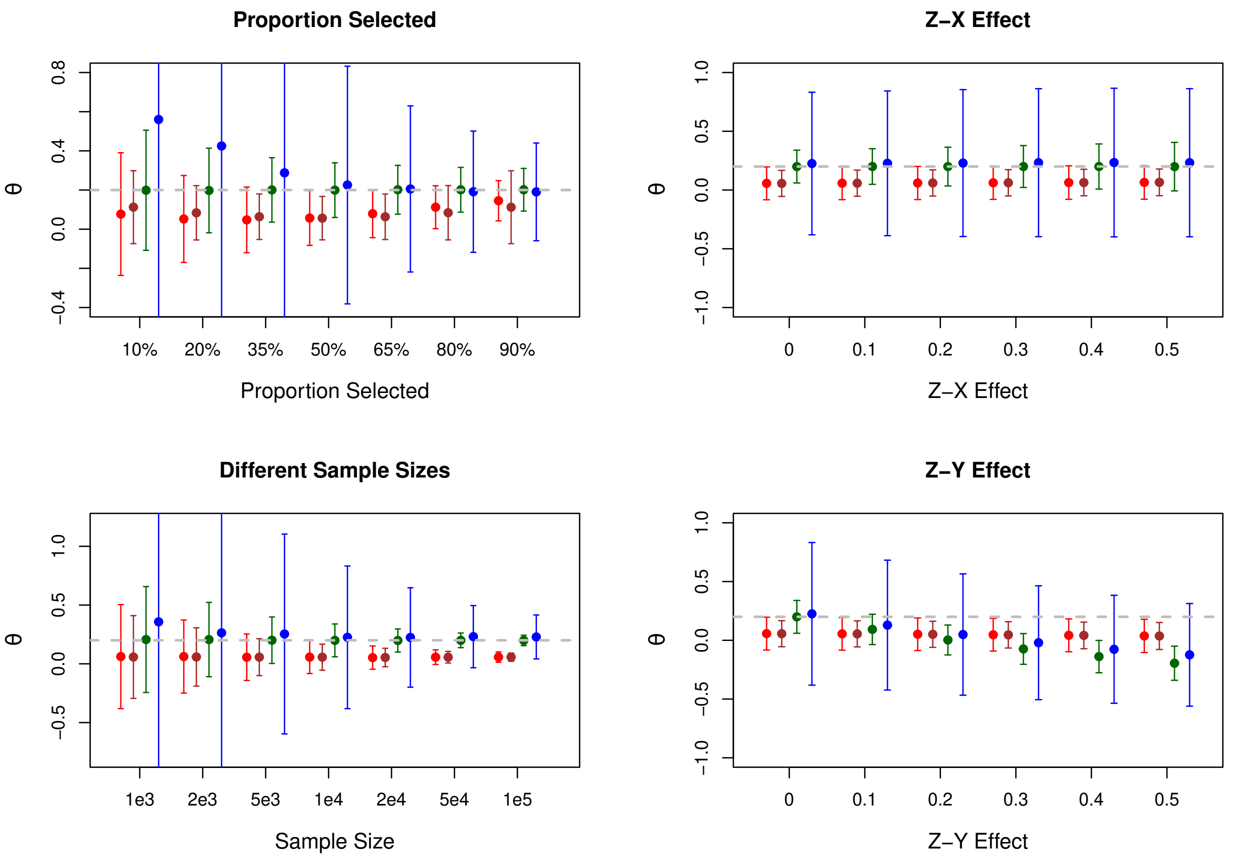


**FIGURE S5** Performance of the various selection bias adjustment methods in MR analyses with a varying proportion of missing data (top left), an added $Z-X$ effect (top right), different sample sizes (bottom left), or an added $Z-Y$ effect (bottom right).

The results of these additional simulations are summarized in Figure S5. To improve the presentation of our results, we have reported median (and not mean) causal effect estimates and computed the averaged confidence intervals using the median (and not mean) standard errors for each method.

Varying the proportion of individuals with fully observed data had a similar impact in MR simulations as in regression-based ones: smaller proportions meant that inference was based on fewer individuals, hence the precision of all methods was reduced. This was more prominent for the TTW method, whose precision was already quite low in the baseline simulation where 50% of all individuals had fully observed data. With a high proportion of missing values, MR analyses would have to be conducted in a sample of only a few thousand individuals, and the use of a method with high in-built uncertainty, such as TTW, seems infeasible in these settings. On the other hand, confidence intervals produced by Heckman's method had comparable width to those from complete-case analysis or IPW.

Similar results were obtained in simulations with different sample sizes. When the sample size of the MR analysis was small, e.g. a few thousand individuals) the various methods had substantial uncertainty in their causal effect estimates. Again, this was more problematic for the TTW method due to its lower overall precision. The complete-case, IPW and Heckman methods had comparable standard errors. On the other hand, with larger sample sizes, the performance of all methods improved.

Unlike the regression-based simulations, a $Z - X$ effect does constitute a violation of the exclusion restriction for $Z$ because the MR exposure acts as an "outcome" for the purpose of estimating the denominator of the Wald ratio. However, inducing a direct $Z - X$ effect did not seem to have a substantial impact on IVsel estimates in our simulations. Note that a $Z - X$ effect violates the exclusion restriction in both the $G - X$ and the $G - Y$ regression analyses (since $Z \not\perp\!\!\!\perp Y|G$), inducing bias in both. Our results in Figure S5 suggest that these two biases may cancel out when computing the Wald ratio estimate.

Finally, with a direct effect between the instrument $Z$ and the outcome $Y$, the second IV assumption for $Z$ was violated and bias was induced. The pattern of bias was similar to that observed in Figure S2 for regression-based analyses, though its magnitude was somewhat weaker. Again, this means that $Z - Y$ effects can devalidate the results of the IVsel methods and this should be kept in mind when deciding which variables to use as instruments for selection. Thankfully, it may be possible to detect such effects in some applications. For example, consider an MR analysis where the instrument for selection is genetic. With access to data from an outcome GWAS, the researchers can investigate whether the instrument for selection $Z$ correlates with the outcome conditional on any instruments for inference $G$, by looking at the associations between the genetic variants that constitute $Z$ and the outcome in the GWAS. Note that the outcome GWAS will itself be affected by the missing data, so this idea comes with no theoretical guarantees of validity, but it could be an informal heuristic for detecting variants in $Z$ that are strongly associated with the outcome and removing them from the instrument for selection.

## Empirical Power of IVsel Methods

In addition to the previous scenarios, we also conducted an MR simulation varying the value of the exposure-outcome causal effect $\theta$ in order to study the power of the IVsel methods to detect a non-null causal effect. Specifically, we considered the values $\theta = 0, 0.05, 0.1, 0.15, 0.2, 0.25, 0.3$ for the causal effect. Once again, this was done only for the case of a one-sample MR analysis with a single instrument for inference, where both the exposure and the outcome have missing data.

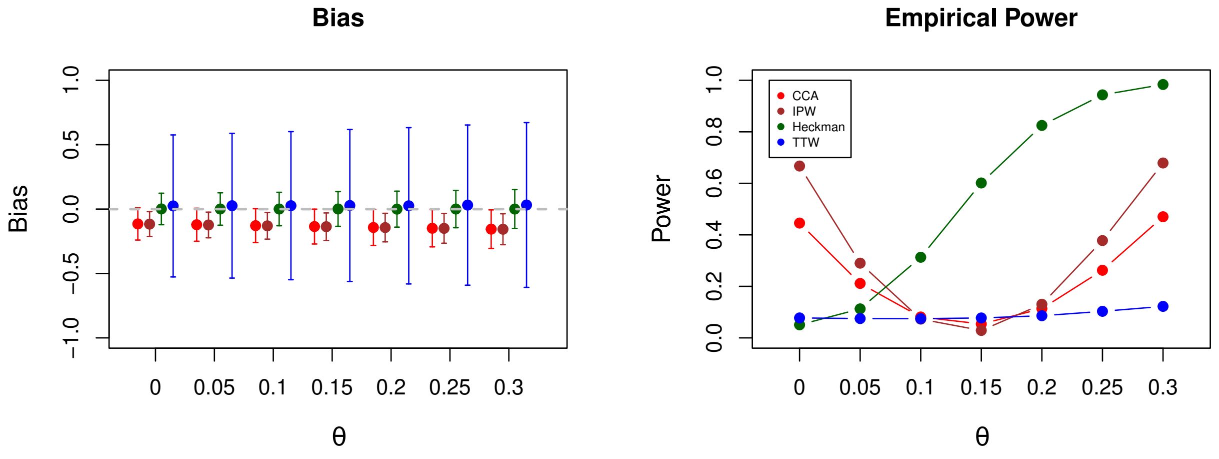


**FIGURE S6** Performance of the various selection bias adjustment methods in MR analyses with different values of the causal effect $\theta$, in terms of causal effect estimation (left) and empirical power to reject the causal null hypothesis (right).

Figure S6 illustrates the results of this simulation. We plot median causal effect estimates (left) and the empirical power of each method to reject the causal null hypothesis (right). The results suggest that varying the value of the causal effect parameter had little impact on the performance of the various methods. However, the difference in the power of the two IVsel methods was considerable. Heckman's method had 60.1% empirical power to detect an effect of 0.15, 82.5% power to detect an effect of 0.2 and 94.3% power to detect an effect of 0.25. On the other hand, the TTW method struggled for power and only had 10.3% power to detect an effect of 0.25. It is worth mentioning that these results concern an MR analysis with a sample size of $N = 10000$ (with approximately half of those individuals having missing data), therefore low power is not unexpected. Nevertheless, the power of TTW is considerably lower than that of the Heckman method, and this should be taken into account when deciding whether to use it in applications.

## MR with a Misspecified First-stage Regression Model

Finally we discuss the impact of model misspecification in the first-stage regression on the performance of selection bias adjustment methods. In particular, we considered the setting where a linear model is assumed for the $G - X$ association, while the true association is non-linear. [14] reported that such misspecification does not introduce bias in a one-sample MR analysis or in a two-sample analysis where the two samples come from the same population. However, in a two-sample analysis where the two samples come from different populations, the misspecification can be a cause of concern.

Here, we aimed to extend the literature results and assess whether the IVsel methods assuming a linear $G - X$ association are indeed robust to model misspecification in the first-stage regression. With that in mind, we implemented six simulation scenarios:

1. One-sample MR, linear first stage.
2. One-sample MR, non-linear first stage.
3. Two-sample MR, same population, linear first stage.
4. Two-sample MR, same population, non-linear first stage.
5. Two-sample MR, different populations, linear first stage.
6. Two-sample MR, different populations, non-linear first stage.

For simplicity, we used a single instrument for inference in this simulation and assumed that missingness was only affected by the outcome $Y$. In simulations with a linear first stage, exposure values were generated according to the equation

$$X = \beta_X G + \gamma_X U + \epsilon_X$$

In scenarios with a non-linear first stage, exposure values were generated according to the equation

$$X = \beta_X G + \beta_{X_2} G^2 + \gamma_X U + \epsilon_X$$

adding a quadratic effect of the instrument $G$ on exposure. Values for $G$ were generated from a $N(0, 1)$ distribution by default. In the last two scenarios where there were two distinct populations, we generated genetic instrument measurements from a $N(0, 1)$ distribution for the first sample and from a $t(4)$ distribution for the second sample. The value of the additional parameter $\beta_{X_2}$ was set to 0.5, representing a fairly strong non-linear effect, to better demonstrate the impact of model misspecification. The true causal effect was set to $\theta = 0.2$.

TABLE S6 HERE

Results are summarized in Table S6. For simulations with a linear first-stage model and no population differences, the performance of the various methods has already been reported in Table 3 and is repeated here. The two-sample MR simulation with different populations and a linear first-stage regression suggests that population differences only had a small impact on this set of simulations. The CCA and IPW methods were still biased, but their bias was similar to that in simulations without population differences, suggesting that it is due to missing data. An increase in the precision of causal effect estimates was observed for all methods.

Our goal here was to compare the performance of the various methods between simulations with a linear and a non-linear first-stage model. For one-sample MR, this comparison suggested that the TTW method was quite sensitive to misspecification of the first-stage regression. Heckman's algorithm and complete-case analysis were affected to a lesser extent (for CCA the overall bias was smaller for a misspecified first-stage model because bias due to misspecification and bias due to missing data acted in opposite directions here). Oracle estimates were unaffected by the misspecification, as suggested in the literature [14], and so were the IPW estimates.

For two-sample MR with data from the same population, the effect of non-linearity in the first-stage regression was similar to that in one-sample MR. However, for two-sample MR with data from different populations, the effect was more pronounced.

TTW was again the most impacted method, with causal effect estimates taking rather extreme values in some iterations. Heckman's method and CCA were also substantially impacted, while oracle estimates were biased but to a smaller degree. Interestingly, the combination of population differences and misspecification in the weighting model had a strong effect on the performance of IPW. The empirical standard deviation of estimates was larger for all methods, while their coverage was also compromised.

The scope of the current simulation was quite limited, as we only considered one form of first-stage model misspecification (a quadratic term for $G$ that is present in the data-generating model but is omitted from the selection model) and one way of generating data from different populations (genetic data simulated from normal and $t(4)$ distributions for the two populations respectively). Therefore we cannot claim our results to be representative of model misspecification and population differences in general and more research in that direction is needed. However, our simulation suggests that first-stage misspecification and population differences are more concerning when acting simultaneously: when only one of these issues was present, the bias of most methods was fairly small in absolute value. An exception occurred for the TTW method, which was more sensitive to misspecification of the first-stage regression than other methods.

## ADDITIONAL RESULTS FROM THE REAL-DATA APPLICATION

Here, we report additional diagnostics and results from our real-data application. In the main part of our manuscript, we focused on the effects of BMI on smoking status and smoking frequency. Here, we expand this analysis in a number of ways. First, we provide more details on the construction of the polygenic risk score for ALSPAC participation. Second, we report numerical results for the two smoking outcomes, to complement the plot in Figure 3, as well as a sensitivity analysis for the number of cigarettes smoked, conducted using Poisson regression. Third, we consider the effects of BMI on two additional behavioral outcomes whose measurement is often subject to selection bias, namely self-harm and depression status. And fourth, we report measures of instrument strength for each instrument for selection.

### Construction of the Risk Score for ALSPAC Participation

Here, we provide more details on the construction of the polygenic risk score for ALSPAC participation. The construction was based on previous work by[15], who investigated genetic associations with participation in ALSPAC. We used data from their work, kindly provided to us by the authors of the study. We focused on overall children participation in order to construct the score, since our MR analyses use data from both clinic visits (BMI) and questionnaires (smoking traits).

Only nine genetic variants were identified as having a GWAS-significant association with overall children participation[15]. These variants were all in high LD ($> 99\%$) and were located in the gene region *BDKRB1* in Chromosome 14. The leading variant was rs28631073. Using only that one variant would produce a rather weak instrument for selection. Therefore, we decided to create a more inclusive polygenic score for ALSPAC participation by relaxing the threshold for inclusion of genetic variants into the risk score. Specifically, we included all variants whose p-value of association with ALSPAC participation was lower than $10^{-4}$. This yielded a total of 1272 variants. The number reduced to 1100 after quality control (discarding non-replacement alleles, triallelic SNPs and variants with minor allele frequency below 1%). We then implemented LD-pruning at a correlation threshold of 0.1 to remove correlated variants; this reduced the number of variants to 178.

We assessed potential violations of the IV assumptions by examining the associations of variants in our polygenic score with BMI, smoking status and the number of cigarettes smoked per day. The associations were quite weak, and in fact none of the 178 variants was associated with any of the three traits after applying a correction for multiple testing. Therefore, all 178 variants were suitable for inclusion into the risk score.

Our PRS was constructed using the associations of the 178 variants with ALSPAC participation as weights. We acknowledge the potential for winner's curse bias to have affected the construction of our risk score, unlike the scores constructed using variants from the UK Biobank. However, this is a price one may have to pay when using genetic instruments for selection. Factors that affect participation in ALSPAC are slightly different to factors affecting participation in UK Biobank or other datasets, so identifying variants for ALSPAC participation based on patterns of participation in other datasets will also be subject to biases. Accordingly, the risk score for participation constructed using ALSPAC data was a much stronger instrument for selection compared to the two risk scores based on the UK Biobank, as we discuss in the next subsections.

## Effects of BMI on Smoking Traits Revisited

Here, we provide numerical results for the effects of BMI on smoking traits; these are reported in Table S7. Figure 3 was created based on these results. As we have already discussed, results obtained from the IVsel methods agree with those from complete-case analysis and IPW in suggesting a causal effect of BMI on number of cigarettes smoked per day, but no effect on smoking status.

TABLE S7 HERE

The MR analysis for the number of cigarettes smoked per day was conducted using linear regression, in order to demonstrate the use of the various selection bias adjustment methods. However, it is also possible (and indeed more reasonable) to model the number of cigarettes smoked per day using Poisson regression. Hence, we modified the MR analyses reported in the main part of the paper by estimating SNP-outcome associations from Poisson regression instead of linear regression, and report Wald ratios, model-based standard errors and 95% confidence intervals for each method in Table S8. Results are not reported for Heckman's sample selection method, because the implementation of the method in R does not support a Poisson model. A graphical illustration of the results is provided in Figure S7. Briefly, the results obtained using the Poisson regression model agree with those from linear regression in advocating a risk-increasing effect of BMI on smoking frequency, although again the TTW method is underpowered and produces wide confidence intervals.

TABLE S8 HERE

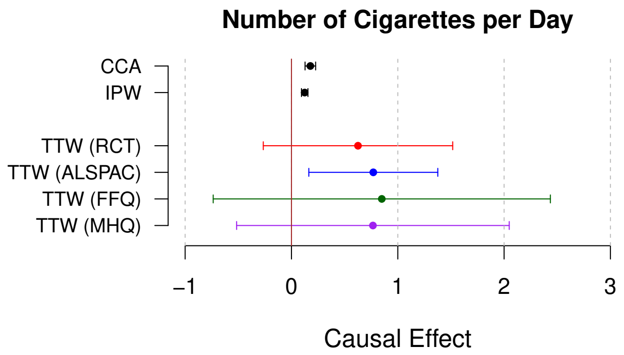


**FIGURE S7** MR estimates of the effect of BMI on the number of cigarettes smoked per day, obtained from ALSPAC data using Poisson regression to model the number of cigarettes smoked and various methods to adjust for missing data. Colors indicate which instrument for selection is used each time (red: RCT for participation, blue: ALSPAC risk score, green: SNPs associated with FFQ completion in UK Biobank, purple: SNPs associated with MHQ completion in UK Biobank, black: method requires no instrument for selection).

## Effects of BMI on Self-Harm and Depression Status

In addition to our analysis about the effects of BMI on smoking traits, we also considered two additional behavioral outcomes, these being self-harm and depression status. Both traits are likely to be affected by selection bias due to missing data, since study participants are often reluctant to disclose personal information about their mental health.

Data on self-harm and depression status were obtained from ALSPAC. Self-harm was self-reported by participants as part of the "It's all about you" questionnaire (age 20+), while depression status was assessed during the TF4 clinic visit (age 18). Both were reported as binary variables. We estimated the effects of BMI on these traits using a Wald ratio estimate and the same polygenic risk score for BMI as in our analyses for smoking outcomes. We then implemented the four selection bias adjustment methods to assess whether our analyses may be biased due to missing data. Results are reported in Table S9 and in graphical form in Figure S8.

TABLE S9 HERE

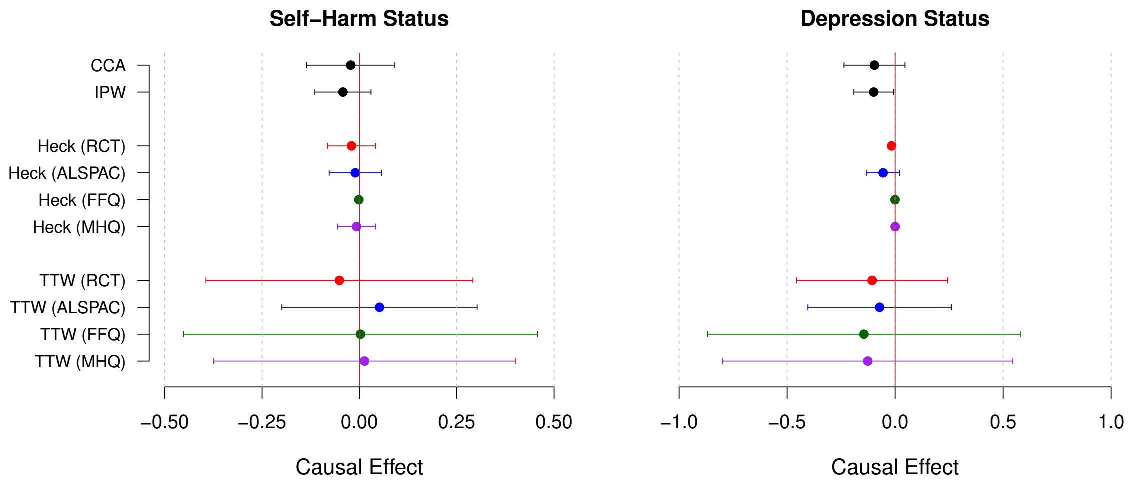


**FIGURE S8** MR estimates of the effect of BMI on self-harm (left) and depression status (right), obtained from ALSPAC data using various methods to adjust for missing data in BMI and the two outcomes. Colors indicate which instrument for selection is used each time (red: RCT for participation, blue: ALSPAC risk score, green: SNPs associated with FFQ completion in UK Biobank, purple: SNPs associated with MHQ completion in UK Biobank, black: method requires no instrument for selection).

Our analysis did not find evidence of a causal effect of BMI on self-harm and this was the case for all four selection bias adjustment methods. For depression status, IPW suggested a risk-decreasing effect, but that effect was quite weak and would not survive a correction for multiple testing. The other methods did not find evidence of a causal effect.

Once again, point estimates from the different methods were in agreement, and IVsel estimates using different instruments were also quite similar. The TTW method again produced estimates with high uncertainty compared to other methods.

Some implementations of Heckman's method suffered from collinearity, resulting in infinite standard error estimates. This was the case for the FFQ instrument in the self-harm MR analysis and for the FFQ, MHQ and RCT instruments in the MR analysis for depression status. For these analyses, we have only plotted the point estimates in Figure S8. In our experience, this performance of Heckman's method tends to occur in binary-outcome analyses with small sample sizes and weak instruments. The prevalence of each outcome may also influence the method's performance: only 11% of genotyped ALSPAC participants who attended the clinic visit exhibited depression symptoms, compared to 60% for smoking (for which Heckman's method produced reasonable standard error estimates).

Comparing our results to the relevant literature,[16] has investigated the effects of genetically elevated BMI on the odds of individuals engaging in self-harm. The authors of that study were unable to identify a causal relationship, although a risk-increasing effect of extreme BMI on self-harm was observed, possibly suggesting that the relationship between BMI and self-harm is non-linear. Our findings are in agreement with the null effect reported in that paper, and the consistent results among different methods suggest that this null finding is unlikely to be due to selection bias. Concerning the relationship between BMI and depression status, the situation is less clear in the literature, with some authors suggesting no causal relationship[17,18] and others reporting a risk-increasing effect[19]. Our results in this applied analysis support a null effect, although as with smoking traits, we should acknowledge that our analyses were performed on fairly young individuals, that the sample size we used was much smaller than that used by other studies in the literature, and that our approach here cannot account for potential non-linearity in the BMI-depression effect.

## Instrument Strength

In the main part of our manuscript, we investigated the effect of instrument strength on the performance of IVsel methods, and illustrated the benefits of using strong instruments when implementing the methods in practice. Here, we report some measures of instrument strength for the four instruments used in our real-data application. In particular, we report the values of the $R^2$ and F statistics, which are commonly used to assess instrument strength. The $R^2$ statistic quantifies the proportion of variation in the trait studied (here, missingness) that is explained by each instrument. The F statistic is routinely used as a measure of instrument strength in instrumental variable analysis, with a common rule-of-thumb suggesting that an F statistic value lower than 10 indicates a weak instrument.

$R^2$ and F statistics for the association of the four instruments with missingness in BMI and the various outcomes were computed from linear regression models with robust standard errors. In addition, we computed the proportion of missing values for each trait, as well as the prevalence among observed values for the three binary traits. These figures are reported in Table S10. We use “RCT" to denote the non-genetic instrument constructed from the RCT for questionnaire completion, “ALSPAC" to denote the polygenic risk score for ALSPAC participation, “FFQ" for the PRS constructed using variants associated with participation in the Food Frequency Questionnaire in UK Biobank and “MHQ" for the PRS constructed from SNPs associated with completion of the Mental Health Questionnaire.

TABLE S10 HERE

The ALSPAC-derived polygenic score was clearly the strongest instrument, explaining about 3.5% of variation in selection for smoking traits and self-harm and an even higher proportion of variation in selection for depression and BMI. F statistics computed using this instrument were much higher than 10. Accordingly, in previous sections, IVsel estimates computed using the ALSPAC PRS as an instrument were subject to lower uncertainty than for the other three instruments.

The RCT, FFQ and MHQ instruments were all quite weak. For the RCT and FFQ instruments, the F statistics of association with selection in all five traits were below 10. The MHQ instrument exhibited acceptable association with selection for smoking traits and self-harm, but low association with selection for BMI and self-harm. The BMI results were particularly concerning, since BMI was used as a risk factor in all our MR analyses. Likewise, the values of the $R^2$ statistic were lower than 0.3% for the MHQ instrument and all traits, and lower than 0.1% for the RCT and FFQ instruments.

Missing data were common for all traits, with about 60% of participants having missing data for smoking status, number of cigarettes and self-harm; the three traits were recorded as part of the same questionnaire, hence they share similar missingness patterns. BMI and depression status also shared similar missingness patterns, since they were recorded as part of the “TF4" clinic visit. These two traits had missing values for approximately half the participants that passed our inclusion criteria (genotyped with recorded maternal traits).

## References


1. Ven V. dWPMM, Van Praag BMS. The demand for deductibles in private health insurance: A probit model with sample selection. *Journal of Econometrics* 1981; 17(2): 229-252. doi: 10.1016/0304-4076(81)90028-2

2. Terza JV. Estimating count data models with endogenous switching: Sample selection and endogenous treatment effects. *Journal of Econometrics* 1998; 84(1): 129-154. doi: 10.1016/S0304-4076(97)00082-1

3. Marchenko YV, Genton MG. A Heckman Selection-t Model. *Journal of the American Statistical Association* 2012; 107(497): 304-317. doi: 10.1080/01621459.2012.656011

4. Newey WK, Powell JL, Walker JR. Semiparametric Estimation of Selection Models: Some Empirical Results. *The American Economic Review* 1990; 80(2): 324–328. doi: 10.2307/2006593

5. Ahn H, Powell JL. Semiparametric estimation of censored selection models with a nonparametric selection mechanism. *Journal of Econometrics* 1993; 58(1): 3-29. doi: 10.1016/0304-4076(93)90111-H

6. Das M, Newey WK, Vella F. Nonparametric Estimation of Sample Selection Models. *The Review of Economic Studies* 2003; 70(1): 33–58. doi: 10.2307/3648610

7. Tchetgen Tchetgen EJ, Wirth KE. A general instrumental variable framework for regression analysis with outcome missing not at random. *Biometrics* 2017; 73(4): 1123-1131. doi: https://doi.org/10.1111/biom.12670

8. Sun B, Liu L, Miao W, Wirth K, Robins J, Tchetgen Tchetgen EJ. Semiparametric Estimation with Data Missing Not at Random Using an Instrumental Variable. *Statistica Sinica* 2018; 28(4): 1965–1983. doi: 10.5705/ss.202016.0324

9. Lee DS. Training, Wages, and Sample Selection: Estimating Sharp Bounds on Treatment Effects. *The Review of Economic Studies* 2009; 76(3): 1071-1102. doi: 10.1111/j.1467-937X.2009.00536.x

10. Marden JR, Wang L, Tchetgen Tchetgen EJ, Walter S, Glymour MM, Wirth KE. Implementation of Instrumental Variable Bounds for Data Missing Not at Random. *Epidemiology* 2018; 29(3): 364-368. doi: 10.1097/EDE.0000000000000811

11. Honoré BE, Hu L. Selection Without Exclusion. *Econometrica* 2020; 88(3): 1007-1029. doi: https://doi.org/10.3982/ECTA16481

12. Jiang Z, Ding P. The directions of selection bias. *Statistics & Probability Letters* 2017; 125: 104-109. doi: https://doi.org/10.1016/j.spl.2017.01.022

13. Shahar DJ, Shahar E. A Theorem at the Core of Colliding Bias. *The International Journal of Biostatistics* 2017; 13(1): 20160055. doi: 10.1515/ijb-2016-0055

14. Zhao Q, Wang J, Spiller W, Bowden J, Small DS. Two-Sample Instrumental Variable Analyses Using Heterogeneous Samples. *Statistical Science* 2019; 34(2): 317 – 333. doi: 10.1214/18-STS692

15. Taylor AE, Jones HJ, Sallis H, et al. Exploring the association of genetic factors with participation in the Avon Longitudinal Study of Parents and Children. *International Journal of Epidemiology* 2018; 47(4): 1207-1216. doi: 10.1093/ije/dyy060

16. Lim KX, Rijsdijk F, Hagenaars SP, et al. Studying individual risk factors for self-harm in the UK Biobank: A polygenic scoring and Mendelian randomisation study. *PLOS Medicine* 2020; 17(6): 1-21. doi: 10.1371/journal.pmed.1003137

17. Walter S, Kubzansky LD, Koenen KC, et al. Revisiting mendelian randomization studies of the effect of body mass index on depression. *American Journal of Medical Genetics Part B: Neuropsychiatric Genetics* 2015; 168(2): 108-115. doi: https://doi.org/10.1002/ajmg.b.32286

18. Howe LJ, Nivard MG, Morris TT, et al. Within-sibship GWAS improve estimates of direct genetic effects. *bioRxiv* 2021. doi: 10.1101/2021.03.05.433935

19. Tyrrell J, Mulugeta A, Wood AR, et al. Using genetics to understand the causal influence of higher BMI on depression. *International Journal of Epidemiology* 2018; 48(3): 834-848. doi: 10.1093/ije/dyy223

**TABLE S1** Performance of the various selection bias adjustment methods in regression analyses, for various simulation scenarios and $\beta = 0$.

| Method | Mean | Emp SD | StdErr | Type I | Mean | Emp SD | StdErr | Type I |
|---|---|---|---|---|---|---|---|---|
| | Binary $Z$ | | | | Binary $X$ | | | |
| CCA | -0.050 | 0.014 | 0.014 | 0.942 | -0.103 | 0.028 | 0.028 | 0.951 |
| IPW | -0.050 | 0.011 | 0.011 | 0.997 | -0.103 | 0.020 | 0.021 | 0.999 |
| Heckman | 0.000 | 0.022 | 0.022 | 0.050 | 0.000 | 0.046 | 0.046 | 0.047 |
| TTW | 0.002 | 0.046 | 0.046 | 0.050 | 0.008 | 0.175 | 0.175 | 0.051 |
| Oracle | 0.000 | 0.010 | 0.010 | 0.050 | 0.000 | 0.020 | 0.020 | 0.050 |
| | Binary Outcome | | | | Discrete Outcome | | | |
| CCA | -0.016 | 0.006 | 0.006 | 0.831 | -0.025 | 0.008 | 0.008 | 0.866 |
| IPW | -0.104 | 0.024 | 0.024 | 0.990 | -0.026 | 0.006 | 0.007 | 0.975 |
| Heckman | -0.002 | 0.032 | 0.032 | 0.056 | — | — | — | — |
| TTW | 0.006 | 0.119 | 0.112 | 0.083 | 0.001 | 0.024 | 0.025 | 0.046 |
| Oracle | 0.000 | 0.004 | 0.004 | 0.050 | 0.000 | 0.006 | 0.006 | 0.051 |
| | No $X - R$ Effect | | | | No $Y - R$ Effect (MAR) | | | |
| CCA | 0.000 | 0.013 | 0.013 | 0.048 | 0.000 | 0.015 | 0.015 | 0.051 |
| IPW | 0.000 | 0.009 | 0.010 | 0.036 | -0.000 | 0.012 | 0.012 | 0.051 |
| Heckman | 0.000 | 0.014 | 0.014 | 0.048 | 0.000 | 0.029 | 0.029 | 0.046 |
| TTW | 0.000 | 0.079 | 0.077 | 0.054 | 0.000 | 0.041 | 0.041 | 0.050 |
| Oracle | 0.000 | 0.010 | 0.010 | 0.047 | 0.000 | 0.010 | 0.010 | 0.049 |
| | Added $Z - X$ Effect | | | | Added $Z - Y$ Effect | | | |
| CCA | -0.061 | 0.014 | 0.014 | 0.995 | -0.065 | 0.015 | 0.015 | 0.993 |
| IPW | -0.061 | 0.011 | 0.011 | 1.000 | -0.065 | 0.011 | 0.011 | 1.000 |
| Heckman | -0.001 | 0.026 | 0.026 | 0.051 | -0.289 | 0.023 | 0.023 | 1.000 |
| TTW | 0.002 | 0.041 | 0.041 | 0.053 | -0.257 | 0.044 | 0.041 | 1.000 |
| Oracle | 0.000 | 0.009 | 0.009 | 0.050 | 0.000 | 0.011 | 0.011 | 0.052 |

**TABLE S2** Performance of the various selection bias adjustment methods in regression analyses with a weak binary instrument for selection.

| Method | $\beta = 0.1$ | | | | | $\beta = 0$ | | | |
|---|---|---|---|---|---|---|---|---|---|
| | **Mean** | **Emp SD** | **StdErr** | **Cover** | **Power** | **Mean** | **Emp SD** | **StdErr** | **Type I** |
| CCA | 0.042 | 0.014 | 0.014 | 0.018 | 0.848 | -0.053 | 0.014 | 0.014 | 0.962 |
| IPW | 0.042 | 0.011 | 0.011 | 0.000 | 0.969 | -0.053 | 0.011 | 0.011 | 0.999 |
| Heckman | 0.097 | 0.065 | 0.066 | 0.960 | 0.315 | -0.003 | 0.061 | 0.062 | 0.038 |
| TTW | 0.104 | 0.078 | 0.077 | 0.948 | 0.273 | 0.002 | 0.076 | 0.075 | 0.048 |
| Oracle | 0.100 | 0.010 | 0.010 | 0.950 | 1.000 | 0.000 | 0.010 | 0.010 | 0.050 |

**TABLE S3** Performance of the various selection bias adjustment methods in simulations where the outcome is confounded with selection instead of directly causing it.

| **Method** | $\beta = 0.1$ | | | | | $\beta = 0$ | | | |
|---|---|---|---|---|---|---|---|---|---|
| | **Mean** | **Emp SD** | **StdErr** | **Cover** | **Power** | **Mean** | **Emp SD** | **StdErr** | **Type I** |
| CCA | 0.075 | 0.016 | 0.016 | 0.666 | 0.996 | -0.025 | 0.016 | 0.016 | 0.341 |
| IPW | 0.075 | 0.012 | 0.012 | 0.455 | 1.000 | -0.025 | 0.012 | 0.012 | 0.545 |
| Heckman | 0.100 | 0.025 | 0.025 | 0.949 | 0.978 | 0.000 | 0.025 | 0.025 | 0.052 |
| TTW | 0.102 | 0.052 | 0.052 | 0.950 | 0.496 | 0.001 | 0.052 | 0.052 | 0.050 |
| Oracle | 0.100 | 0.011 | 0.011 | 0.951 | 1.000 | 0.000 | 0.011 | 0.011 | 0.051 |

**TABLE S4** Performance of the various selection bias adjustment methods in simulations with a non-normal outcome and a misspecified outcome model.

| Method | Mean | Emp SD | StdErr | Cover | Power | Mean | Emp SD | StdErr | Type I |
|---|---|---|---|---|---|---|---|---|---|
| | | | | | $t(4)$ distribution | | | | |
| CCA | 0.022 | 0.020 | 0.020 | 0.030 | 0.208 | -0.072 | 0.020 | 0.020 | 0.950 |
| IPW | 0.021 | 0.016 | 0.016 | 0.000 | 0.264 | -0.074 | 0.016 | 0.016 | 0.998 |
| Heckman | 0.100 | 0.033 | 0.033 | 0.956 | 0.871 | 0.000 | 0.031 | 0.031 | 0.047 |
| TTW | 0.114 | 0.064 | 0.063 | 0.941 | 0.444 | 0.013 | 0.065 | 0.064 | 0.055 |
| Oracle | 0.100 | 0.014 | 0.014 | 0.950 | 1.000 | 0.000 | 0.014 | 0.014 | 0.052 |
| | | | | | Log-normal distribution | | | | |
| CCA | 0.033 | 0.017 | 0.017 | 0.028 | 0.489 | -0.061 | 0.017 | 0.017 | 0.940 |
| IPW | 0.049 | 0.011 | 0.012 | 0.004 | 0.980 | -0.046 | 0.011 | 0.011 | 0.988 |
| Heckman | 0.102 | 0.029 | 0.029 | 0.950 | 0.946 | 0.001 | 0.027 | 0.027 | 0.052 |
| TTW | 0.132 | 0.053 | 0.053 | 0.907 | 0.697 | 0.030 | 0.056 | 0.055 | 0.086 |
| Oracle | 0.100 | 0.010 | 0.010 | 0.951 | 1.000 | 0.000 | 0.010 | 0.010 | 0.047 |
| | | | | | Bimodal distribution | | | | |
| CCA | 0.022 | 0.022 | 0.022 | 0.054 | 0.170 | -0.074 | 0.022 | 0.022 | 0.918 |
| IPW | -0.003 | 0.019 | 0.020 | 0.001 | 0.047 | -0.098 | 0.019 | 0.019 | 0.999 |
| Heckman | 0.097 | 0.036 | 0.036 | 0.950 | 0.781 | -0.002 | 0.035 | 0.034 | 0.050 |
| TTW | 0.075 | 0.072 | 0.069 | 0.923 | 0.203 | -0.022 | 0.073 | 0.071 | 0.067 |
| Oracle | 0.100 | 0.018 | 0.018 | 0.949 | 1.000 | 0.000 | 0.018 | 0.018 | 0.051 |

**TABLE S5** Performance of the various selection bias adjustment methods in one-sample Mendelian randomization simulations with multiple genetic instruments for inference, using a summary statistics approach instead of 2SLS.

| Method | $\theta = 0.2$ | | | | | $\theta = 0$ | | | |
|---|---|---|---|---|---|---|---|---|---|
| | **Causal** | **Emp SD** | **StdErr** | **Cover** | **Power** | **Causal** | **Emp SD** | **StdErr** | **Type I** |
| One-sample MR (Summary Statistics) - $Y \to R$ | | | | | | | | | |
| CCA | 0.147 | 0.054 | 0.061 | 0.900 | 0.686 | 0.008 | 0.051 | 0.057 | 0.025 |
| IPW | 0.147 | 0.038 | 0.049 | 0.889 | 0.911 | 0.006 | 0.037 | 0.044 | 0.026 |
| Heckman | 0.207 | 0.059 | 0.067 | 0.975 | 0.901 | 0.010 | 0.055 | 0.061 | 0.030 |
| TTW | 0.231 | 0.338 | 0.368 | 0.968 | 0.076 | 0.004 | 0.299 | 0.320 | 0.040 |
| Oracle | 0.206 | 0.044 | 0.051 | 0.974 | 0.990 | 0.008 | 0.043 | 0.046 | 0.046 |
| One-sample MR (Summary Statistics) - $X \to R$ | | | | | | | | | |
| CCA | 0.273 | 0.058 | 0.068 | 0.860 | 0.994 | 0.009 | 0.057 | 0.061 | 0.035 |
| IPW | 0.276 | 0.056 | 0.067 | 0.845 | 0.996 | 0.009 | 0.057 | 0.061 | 0.036 |
| Heckman | 0.206 | 0.043 | 0.051 | 0.980 | 0.994 | 0.007 | 0.043 | 0.046 | 0.035 |
| TTW | 0.113 | 0.045 | 0.045 | 0.492 | 0.719 | 0.005 | 0.032 | 0.035 | 0.039 |
| Oracle | 0.208 | 0.042 | 0.050 | 0.978 | 0.996 | 0.007 | 0.043 | 0.046 | 0.036 |
| One-sample MR (Summary Statistics) - $X, Y \to R$ | | | | | | | | | |
| CCA | 0.077 | 0.067 | 0.073 | 0.625 | 0.157 | -0.103 | 0.065 | 0.067 | 0.319 |
| IPW | 0.067 | 0.048 | 0.057 | 0.344 | 0.163 | -0.109 | 0.045 | 0.051 | 0.570 |
| Heckman | 0.214 | 0.062 | 0.068 | 0.962 | 0.902 | 0.010 | 0.061 | 0.062 | 0.046 |
| TTW | 0.382 | 0.229 | 0.285 | 0.953 | 0.225 | 0.192 | 0.227 | 0.255 | 0.091 |
| Oracle | 0.208 | 0.043 | 0.051 | 0.976 | 0.991 | 0.007 | 0.042 | 0.046 | 0.034 |

**TABLE S6** Performance of the various selection bias adjustment methods in Mendelian randomization simulations with a misspecified first-stage model.

| **Method** | **Linear 1st Stage** | | | | | **Non-linear 1st Stage** | | | | |
|---|---|---|---|---|---|---|---|---|---|---|
| | **Causal** | **Emp SD** | **StdErr** | **Cover** | **Power** | **Causal** | **Emp SD** | **StdErr** | **Cover** | **Power** |
| One-sample MR | | | | | | | | | | |
| CCA | 0.143 | 0.056 | 0.059 | 0.847 | 0.696 | 0.154 | 0.054 | 0.057 | 0.882 | 0.787 |
| IPW | 0.143 | 0.039 | 0.049 | 0.847 | 0.886 | 0.142 | 0.038 | 0.050 | 0.860 | 0.875 |
| Heckman | 0.199 | 0.061 | 0.065 | 0.962 | 0.882 | 0.209 | 0.060 | 0.063 | 0.958 | 0.920 |
| TTW | 0.203 | 0.343 | 0.340 | 0.947 | 0.094 | 0.545 | 0.361 | 0.319 | 0.782 | 0.416 |
| Oracle | 0.199 | 0.044 | 0.049 | 0.971 | 0.986 | 0.199 | 0.044 | 0.050 | 0.972 | 0.987 |
| Two-sample MR - Same Population | | | | | | | | | | |
| CCA | 0.144 | 0.058 | 0.059 | 0.841 | 0.690 | 0.155 | 0.058 | 0.057 | 0.873 | 0.774 |
| IPW | 0.144 | 0.041 | 0.049 | 0.835 | 0.875 | 0.144 | 0.043 | 0.050 | 0.835 | 0.853 |
| Heckman | 0.200 | 0.064 | 0.065 | 0.950 | 0.873 | 0.211 | 0.067 | 0.063 | 0.932 | 0.906 |
| TTW | 0.213 | 0.344 | 0.340 | 0.946 | 0.101 | 0.551 | 0.368 | 0.319 | 0.773 | 0.415 |
| Oracle | 0.200 | 0.049 | 0.049 | 0.951 | 0.984 | 0.201 | 0.052 | 0.050 | 0.941 | 0.980 |
| Two-sample MR - Different Populations | | | | | | | | | | |
| CCA | 0.144 | 0.042 | 0.042 | 0.738 | 0.936 | 0.191 | 0.834 | 0.049 | 0.365 | 0.780 |
| IPW | 0.144 | 0.030 | 0.035 | 0.676 | 0.995 | 0.024 | 0.762 | 0.216 | 0.895 | 0.083 |
| Heckman | 0.201 | 0.047 | 0.047 | 0.949 | 0.992 | 0.241 | 0.843 | 0.054 | 0.390 | 0.814 |
| TTW | 0.214 | 0.214 | 0.211 | 0.946 | 0.175 | 4.168 | 2.955 | 0.302 | 0.022 | 0.985 |
| Oracle | 0.200 | 0.035 | 0.036 | 0.952 | 1.000 | 0.213 | 0.671 | 0.043 | 0.435 | 0.861 |

**TABLE S7** MR estimates of the effects of BMI on smoking status and number of cigarettes per day in ALSPAC, estimated using various methods and instruments for selection to adjust for missing data.

| Method | Instrument | Smoking Status | | | Number of Cigarettes | | |
|---|---|---|---|---|---|---|---|
| | | **Causal** | **SD** | **95% CI** | **Causal** | **SD** | **95% CI** |
| CCA | — | 0.028 | 0.048 | (-0.065 , 0.122) | 0.226 | 0.087 | ( 0.056 , 0.396) |
| IPW | — | 0.044 | 0.029 | (-0.013 , 0.102) | 0.182 | 0.089 | ( 0.007 , 0.356) |
| Heckman | RCT | 0.005 | 0.030 | (-0.053 , 0.063) | 0.215 | 0.097 | ( 0.026 , 0.405) |
| Heckman | ALSPAC | 0.017 | 0.030 | (-0.041 , 0.075) | 0.227 | 0.087 | ( 0.057 , 0.398) |
| Heckman | FFQ | 0.016 | 0.027 | (-0.037 , 0.069) | 0.206 | 0.088 | ( 0.033 , 0.380) |
| Heckman | MHQ | 0.015 | 0.026 | (-0.037 , 0.067) | 0.204 | 0.087 | ( 0.033 , 0.375) |
| TTW | RCT | -0.039 | 0.144 | (-0.320 , 0.243) | 0.666 | 0.828 | (-0.958 , 2.289) |
| TTW | ALSPAC | -0.043 | 0.118 | (-0.275 , 0.189) | 0.541 | 0.484 | (-0.407 , 1.488) |
| TTW | FFQ | 0.008 | 0.144 | (-0.274 , 0.290) | 1.027 | 1.408 | (-1.732 , 3.786) |
| TTW | MHQ | 0.017 | 0.140 | (-0.257 , 0.291) | 0.830 | 1.143 | (-1.410 , 3.070) |

**TABLE S8** MR estimates of the effects of BMI on number of cigarettes smoked in ALSPAC, estimated using Poisson regression, and various methods and instruments for selection to adjust for missing data. Estimates from Heckman's method are not reported, because the existing software does not support an implementation of the method for Poisson regression.

| **Method** | **Instrument** | **Number of Cigarettes (Poisson)** | | |
|---|---|---|---|---|
| | | **Causal** | **SD** | 95% **CI** |
| CCA | — | 0.178 | 0.025 | ( 0.128 , 0.227) |
| IPW | — | 0.124 | 0.016 | ( 0.094 , 0.155) |
| TTW | RCT | 0.626 | 0.455 | (-0.265 , 1.517) |
| TTW | ALSPAC | 0.770 | 0.309 | ( 0.163 , 1.376) |
| TTW | FFQ | 0.850 | 0.809 | (-0.736 , 2.435) |
| TTW | MHQ | 0.766 | 0.654 | (-0.516 , 2.048) |

**TABLE S9** MR estimates of the effects of BMI on self-harm and depression status in ALSPAC, estimated using various methods and instruments for selection to adjust for missing data.

| **Method** | **Instrument** | **Self-harm** | | | **Depression Status** | | |
|---|---|---|---|---|---|---|---|
| | | **Causal** | **SD** | 95% **CI** | **Causal** | **SD** | 95% **CI** |
| CCA | — | -0.022 | 0.058 | (-0.136 , 0.091) | -0.096 | 0.072 | (-0.237 , 0.046) |
| IPW | — | -0.042 | 0.037 | (-0.114 , 0.030) | -0.099 | 0.047 | (-0.191 , -0.008) |
| Heckman | RCT | -0.020 | 0.031 | (-0.082 , 0.041) | -0.016 | Inf | — |
| Heckman | ALSPAC | -0.011 | 0.034 | (-0.078 , 0.057) | -0.056 | 0.039 | (-0.131 , 0.020) |
| Heckman | FFQ | -0.002 | Inf | — | -0.001 | Inf | — |
| Heckman | MHQ | -0.007 | 0.025 | (-0.056 , 0.041) | 0.000 | Inf | — |
| TTW | RCT | -0.051 | 0.175 | (-0.394 , 0.291) | -0.107 | 0.178 | (-0.456 , 0.243) |
| TTW | ALSPAC | 0.052 | 0.128 | (-0.199 , 0.302) | -0.072 | 0.169 | (-0.404 , 0.260) |
| TTW | FFQ | 0.003 | 0.232 | (-0.452 , 0.458) | -0.144 | 0.369 | (-0.868 , 0.579) |
| TTW | MHQ | 0.013 | 0.198 | (-0.375 , 0.401) | -0.127 | 0.343 | (-0.799 , 0.545) |

**TABLE S10** Instrument strength diagnostics ($R^2$ and $F$ statistics) for the association of the various instruments for selection with BMI, smoking status, number of cigarettes smoked, self-harm and depression status in ALSPAC.

| Instrument | Statistic | BMI | Smoking | Cigarettes | Self-harm | Depression |
|---|---|---|---|---|---|---|
| RCT | $R^2$ | 0.01 % | 0.09 % | 0.07 % | 0.08 % | 0.02 % |
| | $F$ | 0.1 | 4.8 | 4.1 | 4.3 | 0.9 |
| ALSPAC | $R^2$ | 4.53 % | 3.50 % | 3.50 % | 3.59 % | 4.05 % |
| | $F$ | 409.4 | 305.1 | 308.2 | 313.2 | 363.7 |
| FFQ | $R^2$ | 0.02 % | 0.05 % | 0.05 % | 0.06 % | 0.05 % |
| | $F$ | 1.8 | 4.1 | 3.6 | 3.6 | 4.0 |
| MHQ | $R^2$ | 0.08 % | 0.26 % | 0.27 % | 0.28 % | 0.10 % |
| | $F$ | 6.4 | 20.7 | 21.5 | 21.7 | 8.0 |
| % Missing | | 52.6 % | 60.2 % | 60.6 % | 60.0 % | 56.8 % |
| Prevalence | | — | 60.8 % | — | 21.1 % | 11.3 % |
